# The Negative Feedback of the Glutamine/Prostatitis Loop Identified Among 1400 Metabolites and Prostatitis via Mendelian Randomization

**DOI:** 10.1155/mi/9648279

**Published:** 2025-07-15

**Authors:** Yi Wang, Hao Ji, Yingfei Chen, Bingye Zhu, Yongming Peng, Qianwei Xing

**Affiliations:** ^1^Department of Urology, Affiliated Hospital of Nantong University, Nantong 226001, Jiangsu Province, China; ^2^Department of Urology, Shanghai General Hospital, Shanghai Jiao Tong University School of Medicine, Shanghai 200080, China; ^3^Department of Urology, Tumor Hospital Affiliated to Nantong University, Nantong 226001, Jiangsu Province, China; ^4^Department of Urology, Affiliated Nantong Hospital of Shanghai University (The Sixth People's Hospital of Nantong), Nantong 226001, Jiangsu Province, China

**Keywords:** clinical implications, Mendelian randomization, metabolites, negative feedback loop, prostatitis

## Abstract

**Background:** Prostatitis remains a clinically tricky problem due to its enigmatic etiologies, low cure rates, and relatively high recurrence rates. Therefore, we first employed Mendelian randomization to disclose the causal relationships among 1400 metabolites and prostatitis for a better understanding of the etiologies of prostatitis and thus identifying effective therapeutic targets.

**Methods:** Prostatitis or metabolite-related data were derived from the online FinnGen or genome-wide association study (GWAS) Catalog datasets. Two-sample Mendelian randomization was employed, and sensitivity analyses, including heterogeneity, pleiotropy, and leave-one-out analysis, were applied to evaluate its stability.

**Results:** Four potentially metabolic etiologies were identified for prostatitis, including glutamine degradant levels, adenosine 5'-monophosphate (AMP)–inosine 5'-monophosphate (IMP) ratio, glycolithocholate–glycolithocholate sulfate ratio, and AMP–citrate ratio. Therein, genetic susceptibility to the glutamine degradant levels, the AMP–IMP ratio, or the glycolithocholate–glycolithocholate sulfate ratio could decrease, while the AMP to citrate ratio might increase the risks of prostatitis. Moreover, two potential metabolic phenotypes of prostatitis were also identified, containing glutamine degradant levels and histidine betaine (hercynine) levels, indicating that genetic susceptibility to prostatitis could increase the risks of these two metabolites. Interestingly, we unexpectedly identified the negative feedback of the glutamine/prostatitis loop, showing that not only genetic susceptibility to glutamine degradant levels could decrease the risks of prostatitis but also genetic susceptibility to prostatitis could increase the risks of glutamine degradant levels.

**Conclusion:** Four metabolic etiologies, two metabolic phenotypes, and the glutamine/prostatitis negative feedback loop were first identified by us for prostatitis in the European population to better understand its etiologies and offer novel treatment targets.

**Summary**

• We first employed Mendelian randomization to disclose the causal relationships among 1400 metabolites and prostatitis for a better understanding of the etiologies of prostatitis and thus identifying effective therapeutic targets.
• The Mendelian randomization method used in this study could effectively reduce the influence of confounding variables, making our results more reliable.
• Four metabolic etiologies were identified for prostatitis in the European population to better understand the pathogenesis of prostatitis.
• Two metabolic phenotypes were identified for prostatitis in the European population to better understand the potential mechanisms of prostatitis.
• The negative feedback loop of glutamine/prostatitis was unexpectedly discovered in the European population, suggesting glutamine might be a key therapeutic target for prostatitis.

## 1. Introduction

Prostatitis, defined as the swelling or inflammation of the prostate gland, is reported to affect male populations of all ages, with an estimated prevalence rate of 8.2% worldwide [1]. Based on the statistical data, approximately 50% of men may have symptoms related to prostatitis at some point in their lives [2]. Recently, the National Institutes of Health (NIH) classified prostatitis into four types: acute bacterial prostatitis (type I); chronic bacterial prostatitis (type II); chronic prostatitis/chronic pelvic pain syndrome (type III); and asymptomatic prostatitis (type IV) [3]. In clinical practice, prostatitis is mainly diagnosed according to the white blood cell count, expressed prostatic secretion, clinical symptoms, and systematic physical examinations [4]. Currently, available treatments widely used in clinics for prostatitis include anti-inflammatory drugs, antibiotics, α-receptor blockers, hormonal therapy, and so on [5]. Due to the enigmatic etiologies, low cure rates, and relatively high recurrence rates, prostatitis remains a clinically tricky problem [6]. Hence, there is an urgent need to explore the etiologies and thus find novel targets for treating prostatitis.

Metabolites, as the small molecules of the intermediate or final products of metabolic processes, could not only be regulated by a wide range of factors, including genetics, lifestyle, nutrition, diseases, and gut microbiota, but also affect disease risks and offer potentially therapeutic targets [7–9]. Due to the relatively high heritability levels of many metabolites, it was possible to evaluate the roles of metabolites in diseases via Mendelian randomization [10, 11]. Mendelian randomization is an effective approach that reduces the likelihood of most confounding variables when assessing the causal relationships among exposures and outcomes since alleles are randomly allocated at conception [12]. Ran et al. [13] applied Mendelian randomization to reveal the possible causative links among immune cells and chronic obstructive pulmonary disease without reverse causation. Zhang et al. [14] found that major depressive disorder could increase the risk of prostatitis via Mendelian randomization. Therefore, in this article, we also employed Mendelian randomization to disclose the causal relationships among 1400 metabolites and prostatitis for a better understanding of the etiology of prostatitis and providing manageable intervention sites for treatments.

## 2. Materials and Methods

### 2.1. Study Design, Ethical Approval, and Research Checklist

**Figure 1:**
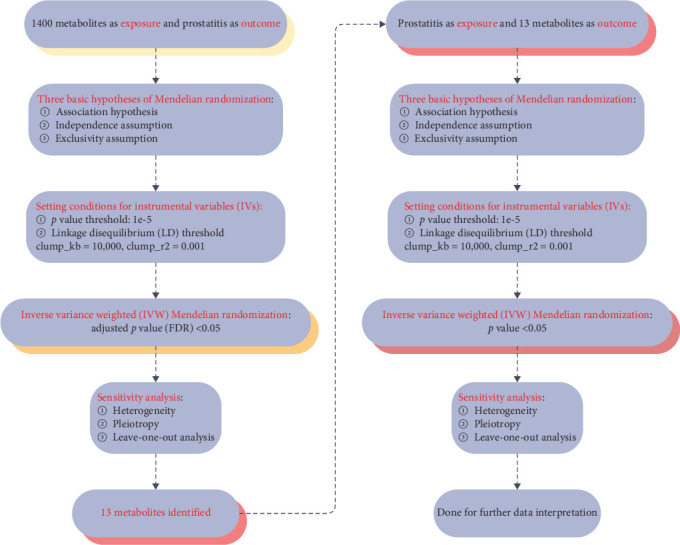
The whole study design.

We used two-sample Mendelian randomization to disclose the causal relationships among 1400 metabolites and prostatitis. Three fundamental Mendelian randomization assumptions, including the correlation assumption, the independence assumption, and the exclusivity assumption [15, 16], formed the foundation of the whole study design, and it was presented in Figure 1. Since the data for this study came from genome-wide association study (GWAS) datasets that were publicly available, ethical approval was therefore not required, and the Strengthening the Reporting of Observational Studies in Epidemiology for Mendelian Randomization (STROBE-MR) checklist was strictly followed in this article [17, 18].

### 2.2. Data Sources

Prostatitis genetic data were derived from the online FinnGen dataset [19] (accessed on 2024-06-01; https://r9.finngen.fi/) with the accession ID of N14_PROSTATITIS and the downloaded link (https://storage.googleapis.com/finngen-public-data-r9/summary_stats/finngen_R9_N14_PROSTATITIS.gz), including 3760 cases and 119,297 controls. Meanwhile, genetic data on metabolites were from the online GWAS Catalog database (accessed on 2024-06-01; https://www.ebi.ac.uk/gwas/), with the GWAS IDs ranging from GCST90199621 to GCST90201020, including 1400 metabolites [20]. All the study subjects involved in this study were European.

### 2.3. Instrumental Variables (IVs) Selection

Single-nucleotide polymorphisms (SNPs) were employed by Mendelian randomization as IVs to disclose the causal relationships among 1400 metabolites and prostatitis. No matter whether 1400 metabolites or prostatitis were exposures, SNPs that met the four criteria listed below were considered IVs: First, the *p* value threshold was set below 1e–5, ensuring the close correlations of IVs. Second, the linkage disequilibrium (LD) thresholds were set as clumped kb = 10000 and *r*2 = 0.001, ensuring the independence of IVs. Third, the PhenoScanner database was applied to systematically screen and remove SNPs associated with confounding factors [21]. Finally, *F*-statistic values were set above 10, avoiding the biases from weak IVs [22].

### 2.4. Sensitivity Analysis

Sensitivity analyses, including heterogeneity, pleiotropy, and leave-one-out analysis, were applied in this article to evaluate the stability of our results. The Cochran's *Q* test was used to assess heterogeneity, and its values of less than 0.05 were considered statistically significant [23]. The MR–Egger regression method was conducted to estimate pleiotropy, and its *p* values of less than 0.05 were deemed statistically significant against the independence assumption of Mendelian randomization [16]. The leave-one-out analysis was performed by eliminating each SNP one at a time to evaluate the robustness of our findings [24].

### 2.5. Statistical Analysis

Two-sample Mendelian randomization was applied in this article to disclose the causal relationships among 1400 metabolites and prostatitis with the help of the R package “TwoSampleMR” and the R version 4.2.1 software (http://www.Rproject.org). A total of five methods were calculated during the analysis, including the inverse variance weighted (IVW) method, the weighted median method, the MR Egger method, the simple mode method, and the weighted mode method. Therein, the IVW method was regarded as the main outcome of this study. In addition, statistically significant differences in the present article were defined as *p* values less than 0.05.

## 3. Results

### 3.1. The Causative Links Among Metabolic Phenotypes' Onsets and Prostatitis

**Figure 2:**
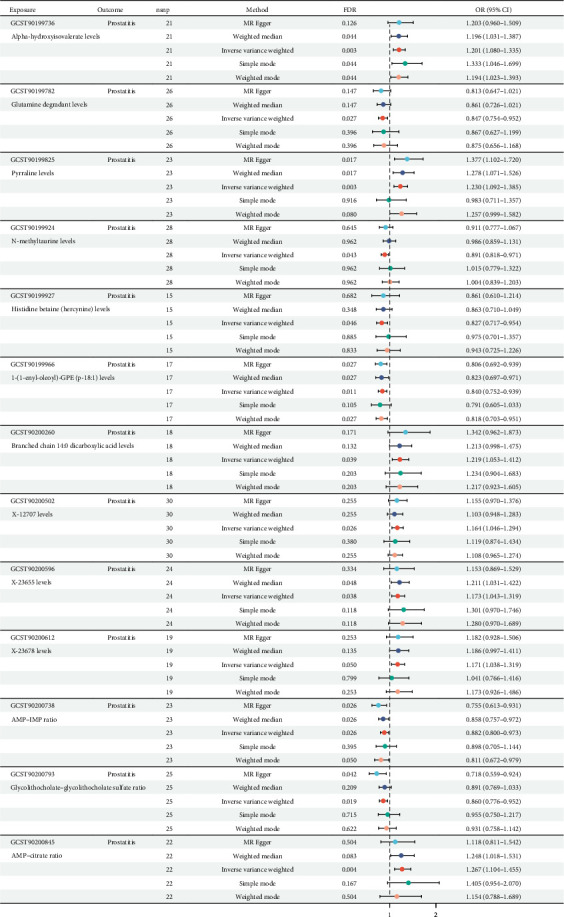
The causative links among metabolic phenotypes' onsets and prostatitis.

We used two-sample Mendelian randomization to disclose the causal relationships among 1400 metabolites and prostatitis, and the related study design is presented in Figure 1. The setting conditions for IVs, the harmonized data, and the results of heterogeneity and pleiotropy for the causality of 1400 metabolites' susceptibilities to prostatitis were detailed in Supporting Information 1: Table S1 and Supporting Information 2: Table S2. After adjusted *p* value (FDR) < 0.05 correction, our results showed that a total of 13 metabolites were found to be significantly linked with prostatitis risks, based on the IVW method (Figure 2). However, nine out of 13 metabolites' results of pleiotropy (*p* values) were less than 0.05, against the independence assumption of Mendelian randomization. Hence, only four metabolites, including glutamine degradant levels (GCST90199782; odds ratio [OR] = 0.847; 95% confidence interval [CI] = 0.754–0.952), adenosine 5'-monophosphate (AMP)–inosine 5'-monophosphate (IMP) ratio (GCST90200738; OR = 0.882; 95% CI = 0.800–0.973), glycolithocholate–glycolithocholate sulfate ratio (GCST90200793; OR = 0.860; 95% CI = 0.776–0.952), and AMP–citrate ratio (GCST90200845; OR = 1.267; 95% CI = 1.104–1.455), were found to be significantly linked with prostatitis risks, based on the IVW method (all FDR < 0.05). Further leave-one-out analysis for the causative links among these four metabolic phenotypes' onsets and prostatitis indicated the stability of our results (Supporting Information 3: Figure S1). All in all, our results found that genetic susceptibility to four metabolic phenotypes, including glutamine degradant levels, AMP–IMP ratio, glycolithocholate–glycolithocholate sulfate ratio, and AMP–citrate ratio, could increase the risks of prostatitis, shedding light on its potential etiologies.

### 3.2. The Causative Links Among Prostatitis and Metabolic Phenotypes

**Figure 3:**
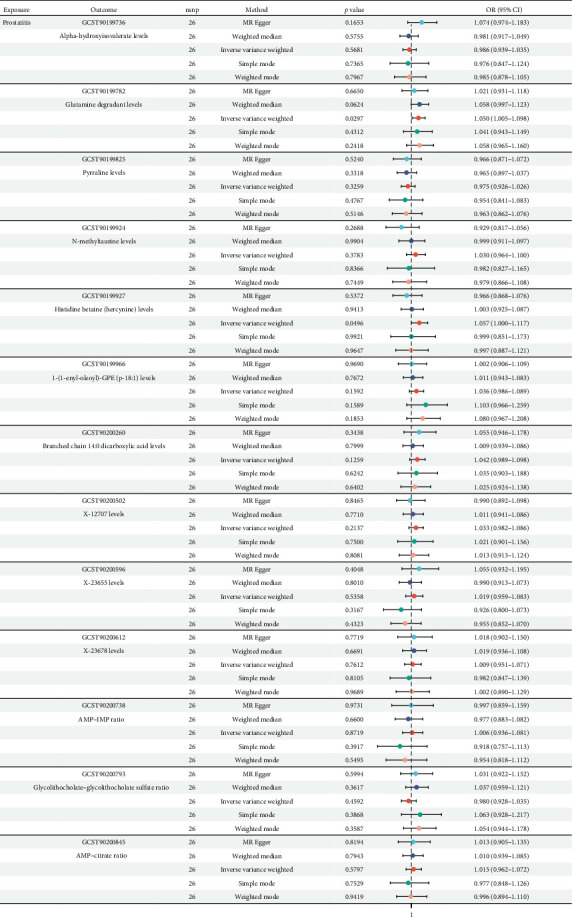
The causative links among prostatitis' onset and metabolic phenotypes.

Further, we used two-sample Mendelian randomization to disclose the causal relationships among prostatitis and 13 metabolites, and the related study design was presented in Figure 1. The setting conditions for IVs, the harmonized data, and the results of heterogeneity and pleiotropy for the causality of prostatitis susceptibility to 13 metabolites were detailed in Supporting Information 4: Table S3 and Supporting Information 5: Table S4. After setting the threshold of *p* value < 0.05, our results showed that prostatitis was found to be significantly linked with a total of two metabolite risks, including glutamine degradant levels (GCST90199782; OR = 1.050; 95% CI = 1.005–1.098) and histidine betaine (hercynine) levels (GCST90199927; OR = 1.057; 95% CI = 1.000–1.117), based on the IVW method (both *p* values < 0.05; Figure 3). Further leave-one-out analysis for the causative links among prostatitis onset and these two metabolic phenotypes indicated the stability of our results (Supporting Information 6: Figure S2). All in all, our results found that genetic susceptibility to prostatitis could increase the risks of two metabolites, including glutamine degradant levels and histidine betaine (hercynine) levels, shedding light on the potential metabolic phenotypes of prostatitis.

### 3.3. The Negative Feedback of the Glutamine/Prostatitis Loop

**Figure 4:**
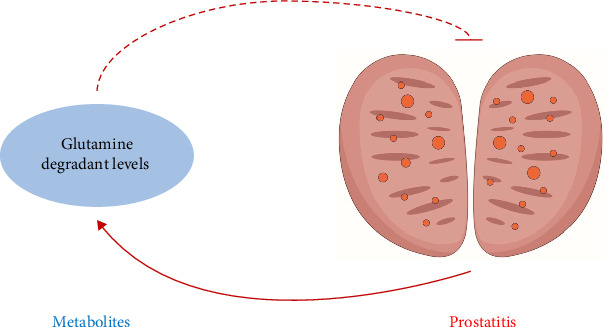
Sketch map for the negative feedback of the glutamine/prostatitis loop, showing that not only genetic susceptibility to glutamine degradant levels could decrease the risks of prostatitis but also genetic susceptibility to prostatitis could increase the risks of glutamine degradant levels.

Interestingly, we noticed that not only genetic susceptibility to glutamine degradant levels could decrease the risks of prostatitis but also genetic susceptibility to prostatitis could increase the risks of glutamine degradant levels. Based on the above-mentioned results, we unexpectedly identified the negative feedback of the glutamine/prostatitis loop, and its sketch map is detailed in Figure 4.

## 4. Discussion

As we know, prostatitis remains a clinically tricky problem due to its enigmatic etiologies, low cure rates, and relatively high recurrence rates [6, 25]. Therefore, there is an urgent need to explore the etiologies and thus find novel targets for treating prostatitis. Meanwhile, metabolites, as the small molecules of the intermediate or final products of metabolic processes, have been found to be significantly related to prostatitis [26, 27]. Hence, in this article, we employed Mendelian randomization to disclose the causal relationships among 1400 metabolites and prostatitis for a better understanding of the etiology of prostatitis and providing manageable intervention sites for treatments.

Based on our results, four potential metabolic etiologies were identified for prostatitis, including glutamine degradant levels, AMP–IMP ratio, glycolithocholate–glycolithocholate sulfate ratio, and AMP–citrate ratio. Therein, genetic susceptibility to the glutamine degradant levels, the AMP–IMP ratio, or the glycolithocholate–glycolithocholate sulfate ratio could decrease, while the AMP–citrate ratio might increase the risks of prostatitis. Moreover, two potential metabolic phenotypes of prostatitis were also identified, containing glutamine degradant levels and histidine betaine (hercynine) levels, indicating that genetic susceptibility to prostatitis could increase the risks of these two metabolites. Interestingly, we unexpectedly identified the negative feedback of the glutamine/prostatitis loop, showing that not only genetic susceptibility to glutamine degradant levels could decrease the risks of prostatitis, but also genetic susceptibility to prostatitis could increase the risks of glutamine degradant levels.

Various risk factors have been revealed in prostatitis by previous articles, including smoking status, sedentary lifestyles, stress, age, and so on [28, 29]. As a powerful tool, Mendelian randomization has been widely applied to identify disease risks by assessing the causal relationships among exposures and outcomes [14, 30, 31]. Yan et al. [31] revealed that COVID-19 infection could increase the risks of herpes simplex virus type-2 infection and decrease the risks of herpes simplex virus type-1 infection. Li et al. [30] showed that genetic susceptibility to platelet crit levels could increase the risks of ischemic stroke, mediated by blood pressure. As for prostatitis, Zhang et al. [14] found that major depressive disorder could increase the risk of prostatitis by means of Mendelian randomization. Based on our results in this article, four potential metabolic etiologies were identified for prostatitis, including glutamine degradant levels, AMP–IMP ratio, glycolithocholate–glycolithocholate sulfate ratio, and AMP–citrate ratio. Glutamine metabolism had previously been found to be involved in the development and progress of both tumor and nontumor diseases [32–34]. The abnormal AMP–IMP ratio was reported to be linked with the purine nucleotide profile of dystrophic muscle in mice, chickens, and humans [35]. As for the glycolithocholate–glycolithocholate sulfate ratio and the AMP–citrate ratio, they had been rarely studied in previous research. Taken together, all of these four metabolites were identified by us as potentially novel etiologies in prostatitis for a better understanding of its mechanisms and providing new treatment targets.

Various phenotypes of prostatitis had been identified in previous studies, including apoptosis, fibrosis, oxidative stress, cell proliferation, cell cycle, and so on [36–38]. Hu et al. [38] shed light on the fact that type III prostatitis could impair erectile function by means of endothelial dysfunction, apoptosis, fibrosis, and oxidative stress in rats' corpus cavernosum. Xu et al. [37] found that overexpressed lncRNA GAS5 was able to inhibit the proliferation of prostatic epithelial cells via regulating COX-2 in prostatitis. He et al. [36] demonstrated that resveratrol was capable of enhancing cell cycle arrest through suppressing C-kit/SCF in rats with chronic prostatitis. Based on our results in this article, two potential metabolic phenotypes of prostatitis were also identified, containing glutamine degradant levels and histidine betaine (hercynine) levels, indicating that genetic susceptibility to prostatitis could increase the risks of these two metabolites. Currently, both of these two metabolic phenotypes have been rarely studied in previously published prostatitis-related articles. Taken together, both glutamine degradant levels and histidine betaine (hercynine) levels were identified by us as novel metabolic phenotypes of prostatitis for better understanding its mechanisms and offering new therapeutic targets.

Negative feedback was commonly present in various diseases, including both tumor and nontumor diseases [39–41]. In tumors, Ecker et al. [42] presented that the development of chronic lymphocytic leukemia was significantly influenced by the regulation of negative feedback in MAPK signaling. Feng et al. [43] shed light on the fact that PTEN and ATF6*α* reciprocal negative feedback regulation accelerated the development of prostate cancer. In nontumor diseases, Xu et al. [44] revealed that DNMT3A and miR-145 had a bidirectional negative feedback loop to control autophagy in cardiac fibroblasts and influence myocardial fibrosis. Yan et al. [45] reported the negative feedback loop of TGF-β1 and JNK-associated leucine zipper protein in the regulation of kidney fibrosis. However, prostatitis-related negative feedback is currently seldom studied after searching in PubMed. Interestingly, in this article, we unexpectedly identified the negative feedback of the glutamine/prostatitis loop, showing that not only genetic susceptibility to glutamine degradant levels could decrease the risks of prostatitis, but also genetic susceptibility to prostatitis could increase the risks of glutamine degradant levels. Obviously, glutamine could serve as a critical therapeutic target for prostatitis, requiring further in vivo and in vitro experimental validation.

A few limitations in our research should never be neglected. First, all the study subjects involved in this study were European. So, our conclusions currently cannot not be applied to other racial groups. Second, a looser threshold of *p* value < 0.05 was set when assessing the causal relationships among prostatitis and 13 metabolites, which might lead to increased false-positive results. Third, the diagnostic criteria for prostatitis in the database were absent, and the additional stratified analysis of the population was not performed due to the absence of key information. Finally, the present manuscript was entirely based on database analysis, without any experimental validation or clinical data, and these jobs shall be conducted in our subsequent articles.

## 5. Conclusions

All in all, four potentially metabolic etiologies were identified by Mendelian randomization for prostatitis in the European population, including glutamine degradant levels, AMP–IMP ratio, glycolithocholate–glycolithocholate sulfate ratio, and AMP–citrate ratio. Moreover, two potential metabolic phenotypes of prostatitis were identified in the European population, containing glutamine degradant levels and histidine betaine (hercynine) levels. Unexpectedly, we also identified the negative feedback of the glutamine/prostatitis loop and found that glutamine might be a key therapeutic target for prostatitis in the European population to better understand its etiology and offer novel therapeutic targets.

## Data Availability

Prostatitis genetic data were derived from the online FinnGen dataset (accessed on 2024-06-01; https://r9.finngen.fi/) with the accession ID of N14_PROSTATITIS and the downloaded link (https://storage.googleapis.com/finngen-public-data-r9/summary_stats/finngen_R9_N14_PROSTATITIS.gz). Genetic data on 1400 metabolites were from the online GWAS Catalog database (accessed on 2024-06-01; https://www.ebi.ac.uk/gwas/), with the GWAS IDs ranging from GCST90199621 to GCST90201020.

## Supporting Information

Supporting Information 1 Table S1. Setting conditions for instrumental variables (IVs) and results of heterogeneity and pleiotropy for the causality of metabolite susceptibility to prostatitis.

Supporting Information 2 Table S2. The harmonized data for the causality of metabolite susceptibility to prostatitis.

Supporting Information 3 Figure S1. Leave-one-out analysis for the causative links among metabolic phenotypes onset and prostatitis.

Supporting Information 4 Table S3. Setting conditions for instrumental variables (IVs) and results of heterogeneity and pleiotropy for the causality of prostatitis susceptibility to metabolites.

Supporting Information 5 Table S4. The harmonized data for the causality of prostatitis susceptibility to metabolites.

Supporting Information 6 Figure S2. Leave-one-out analysis for the causative links among prostatitis onset and metabolic phenotypes.
