# Supplementary material for: The Negative Feedback of the Glutamine/Prostatitis Loop Identified Among 1400 Metabolites and Prostatitis via Mendelian Randomization
Source: Mediators Inflamm. 2025 Jul 15;2025:9648279. doi: 10.1155/mi/9648279 (PMC12283193; doi:10.1155/mi/9648279)
Supplement: Supporting Information 1 — Table S1. Setting conditions for instrumental variables (IVs) and results of heterogeneity and pleiotropy for the causality of metabolite susceptibility to prostatitis. [file 9648279.f1.docx]

**Table S1**. Setting conditions for instrumental variables (IVs) and results of heterogeneity and pleiotropy for the causality of metabolite susceptibility to prostatitis;

| Exposure | Outcome | P-value threshold | Linkage disequilibrium threshold | Heterogeneity (Q_pval) | Pleiotropy (pval) |
| --- | --- | --- | --- | --- | --- |
| GCST90199736  (Alpha-hydroxyisovalerate levels) | finn-b-N14_PROSTATITIS  (Prostatitis) | 1e-5 | clump_kb=10000, clump_r2=0.001 | 0.652 | **0.015** |
| GCST90199782  (Glutamine degradant levels) | finn-b-N14_PROSTATITIS  (Prostatitis) | 1e-5 | clump_kb=10000, clump_r2=0.001 | 0.748 | 0.681 |
| GCST90199825  (Pyrraline levels) | finn-b-N14_PROSTATITIS  (Prostatitis) | 1e-5 | clump_kb=10000, clump_r2=0.001 | 0.547 | **0.014** |
| GCST90199924  (N-methyltaurine levels) | finn-b-N14_PROSTATITIS  (Prostatitis) | 1e-5 | clump_kb=10000, clump_r2=0.001 | 0.709 | **0.012** |
| GCST90199927  (Histidine betaine (hercynine) levels) | finn-b-N14_PROSTATITIS  (Prostatitis) | 1e-5 | clump_kb=10000, clump_r2=0.001 | 0.714 | **0.025** |
| GCST90199966  (1-(1-enyl-oleoyl)-GPE (p-18:1) levels) | finn-b-N14_PROSTATITIS  (Prostatitis) | 1e-5 | clump_kb=10000, clump_r2=0.001 | 0.921 | **0.012** |
| GCST90200260  (Branched chain 14:0 dicarboxylic acid levels) | finn-b-N14_PROSTATITIS  (Prostatitis) | 1e-5 | clump_kb=10000, clump_r2=0.001 | 0.998 | **0.020** |
| GCST90200502  (X-12707 levels) | finn-b-N14_PROSTATITIS  (Prostatitis) | 1e-5 | clump_kb=10000, clump_r2=0.001 | 0.129 | **0.015** |
| GCST90200596  (X-23655 levels) | finn-b-N14_PROSTATITIS  (Prostatitis) | 1e-5 | clump_kb=10000, clump_r2=0.001 | 0.851 | **0.022** |
| GCST90200612  (X-23678 levels) | finn-b-N14_PROSTATITIS  (Prostatitis) | 1e-5 | clump_kb=10000, clump_r2=0.001 | 0.518 | **0.020** |
| GCST90200738  (AMP to IMP ratio) | finn-b-N14_PROSTATITIS  (Prostatitis) | 1e-5 | clump_kb=10000, clump_r2=0.001 | 0.224 | 0.118 |
| GCST90200793  (Glycolithocholate to glycolithocholate sulfate ratio) | finn-b-N14_PROSTATITIS  (Prostatitis) | 1e-5 | clump_kb=10000, clump_r2=0.001 | 0.932 | 0.139 |
| GCST90200845  (AMP to citrate ratio) | finn-b-N14_PROSTATITIS  (Prostatitis) | 1e-5 | clump_kb=10000, clump_r2=0.001 | 0.561 | 0.407 |
