# Supplementary material for: The Negative Feedback of the Glutamine/Prostatitis Loop Identified Among 1400 Metabolites and Prostatitis via Mendelian Randomization
Source: Mediators Inflamm. 2025 Jul 15;2025:9648279. doi: 10.1155/mi/9648279 (PMC12283193; doi:10.1155/mi/9648279)
Supplement: Supporting Information 2 — Table S2. The harmonized data for the causality of metabolite susceptibility to prostatitis. [file 9648279.f2.docx]

**Table S2**. The harmonized data for the causality of metabolite susceptibility to prostatitis;

| GCST90199736 |  |  |  |  |  |  |  |  |  |  |  |
| --- | --- | --- | --- | --- | --- | --- | --- | --- | --- | --- | --- |
| SNP | **effect_allele.exposure** | **other_allele.exposure** | **effect_allele.outcome** | **other_allele.outcome** | **beta.exposure** | **beta.outcome** | **pval.outcome** | **pval.exposure** | **samplesize.exposure** | **R2** | **f** |
| rs10225370 | A | T | A | T | -0.09913 | -0.01375 | 0.659171 | 7.16E-06 | 8208 | 0.002449 | 20.14531 |
| rs10802092 | T | A | T | A | 0.174872 | 0.033788 | 0.165505 | 1.95E-31 | 8208 | 0.016304 | 136.0113 |
| rs10808894 | T | A | T | A | 0.147132 | 0.129946 | 0.012986 | 8.51E-06 | 8208 | 0.002409 | 19.8143 |
| rs112297849 | G | A | G | A | 0.325275 | 0.181395 | 0.126931 | 9.61E-06 | 8208 | 0.002381 | 19.58255 |
| rs114874682 | A | T | A | T | -0.20265 | -0.09241 | 0.106388 | 6.81E-07 | 8208 | 0.002996 | 24.66166 |
| rs117055304 | C | T | C | T | 0.221934 | -0.05862 | 0.50963 | 2.82E-06 | 8208 | 0.002665 | 21.92729 |
| rs142429133 | T | A | T | A | 0.502481 | -0.0052 | 0.981256 | 5.83E-06 | 8208 | 0.002496 | 20.53736 |
| rs165107 | T | G | T | G | -0.1017 | -0.03999 | 0.220007 | 1.51E-06 | 8208 | 0.002811 | 23.13435 |
| rs186454994 | T | C | T | C | 0.313174 | 0.109122 | 0.411041 | 2.60E-06 | 8208 | 0.002684 | 22.08658 |
| rs1891761 | G | A | G | A | -0.07288 | -0.02526 | 0.287904 | 9.69E-07 | 8208 | 0.002914 | 23.98369 |
| rs2393791 | T | C | T | C | -0.11618 | -0.0239 | 0.320878 | 2.37E-14 | 8208 | 0.007041 | 58.18594 |
| rs28456695 | G | C | G | C | 0.220041 | -0.0788 | 0.564262 | 3.22E-06 | 8208 | 0.002634 | 21.67348 |
| rs2927480 | C | G | C | G | -0.0814 | -0.03117 | 0.205539 | 4.64E-07 | 8208 | 0.003086 | 25.40242 |
| rs3802967 | C | T | C | T | -0.25877 | -0.03626 | 0.125189 | 4.35E-73 | 8208 | 0.038312 | 326.9108 |
| rs541290556 | T | C | T | C | -0.2421 | 0.109025 | 0.248745 | 9.90E-06 | 8208 | 0.002374 | 19.52669 |
| rs6085741 | A | C | A | C | 0.104775 | 0.03252 | 0.291494 | 7.08E-08 | 8208 | 0.003526 | 29.03479 |
| rs655558 | T | C | T | C | -0.0695 | 0.033185 | 0.171082 | 5.25E-06 | 8208 | 0.002521 | 20.74048 |
| rs6696772 | C | G | C | G | -0.07943 | -0.02279 | 0.440515 | 8.83E-06 | 8208 | 0.0024 | 19.74337 |
| rs7236320 | A | G | A | G | -0.08209 | -0.02146 | 0.390911 | 3.45E-06 | 8208 | 0.002619 | 21.54595 |
| rs76197763 | G | A | G | A | -0.17783 | -0.04171 | 0.47614 | 7.73E-06 | 8208 | 0.002431 | 19.99769 |
| rs950950 | T | C | T | C | 0.0691 | -0.00943 | 0.69228 | 2.34E-06 | 8208 | 0.002709 | 22.28689 |
| GCST90199782 |  |  |  |  |  |  |  |  |  |  |  |
| SNP | effect_allele.exposure | other_allele.exposure | effect_allele.outcome | other_allele.outcome | beta.exposure | beta.outcome | pval.outcome | pval.exposure | samplesize.exposure | R2 | f |
| rs10222346 | T | C | T | C | 0.063358 | -0.01632 | 0.502545 | 4.24E-06 | 8293 | 0.002544 | 21.14906 |
| rs10774021 | T | C | T | C | 0.147532 | 0.025971 | 0.307537 | 1.11E-27 | 8293 | 0.014134 | 118.8629 |
| rs10830022 | G | C | G | C | -0.06415 | -0.00057 | 0.980994 | 1.51E-06 | 8293 | 0.002782 | 23.12634 |
| rs113157327 | A | G | A | G | 0.074081 | 0.002903 | 0.921891 | 9.38E-06 | 8293 | 0.002362 | 19.62939 |
| rs11780900 | G | A | G | A | 0.073738 | 0.012103 | 0.685021 | 9.46E-06 | 8293 | 0.00236 | 19.61198 |
| rs12611131 | T | C | T | C | 0.102723 | -0.03826 | 0.240107 | 1.78E-06 | 8293 | 0.002744 | 22.8099 |
| rs140369356 | A | G | A | G | -0.18936 | 0.0291 | 0.626511 | 2.99E-06 | 8293 | 0.002625 | 21.81879 |
| rs145062142 | G | C | G | C | -0.20652 | 0.030933 | 0.845457 | 9.61E-06 | 8293 | 0.002356 | 19.58298 |
| rs146767775 | T | G | T | G | -0.49726 | 0.074375 | 0.693172 | 4.81E-17 | 8293 | 0.008419 | 70.39698 |
| rs17279437 | A | G | A | G | 0.238955 | 0.002618 | 0.947285 | 2.80E-29 | 8293 | 0.014988 | 126.1545 |
| rs200231 | T | A | T | A | 0.21855 | -0.0931 | 0.279013 | 2.00E-06 | 8293 | 0.002717 | 22.58506 |
| rs2041517 | C | T | C | T | 0.057761 | -0.00465 | 0.848458 | 9.18E-06 | 8293 | 0.002367 | 19.67036 |
| rs2467862 | G | T | G | T | -0.08051 | 0.023565 | 0.325244 | 1.91E-09 | 8293 | 0.00433 | 36.05345 |
| rs2737700 | G | A | G | A | 0.060247 | -0.00748 | 0.752295 | 2.90E-06 | 8293 | 0.002632 | 21.87732 |
| rs35518298 | T | C | T | C | 0.193234 | -0.03627 | 0.633814 | 7.57E-06 | 8293 | 0.002411 | 20.03989 |
| rs35881890 | A | G | A | G | 0.11164 | -0.05921 | 0.144664 | 6.61E-06 | 8293 | 0.002442 | 20.29726 |
| rs4726088 | A | G | A | G | -0.06166 | 0.026322 | 0.26562 | 4.42E-06 | 8293 | 0.002535 | 21.06891 |
| rs5754226 | G | A | G | A | -0.08605 | 0.034878 | 0.300085 | 1.78E-06 | 8293 | 0.002744 | 22.81583 |
| rs61792192 | C | T | C | T | 0.366127 | 0.385738 | 0.205378 | 8.74E-06 | 8293 | 0.002378 | 19.76318 |
| rs715 | C | T | C | T | -0.15082 | 0.032117 | 0.2103 | 3.73E-27 | 8293 | 0.013851 | 116.4534 |
| rs7163136 | G | A | G | A | -0.11139 | 0.001098 | 0.977038 | 1.91E-06 | 8293 | 0.002727 | 22.67424 |
| rs73260922 | A | G | A | G | 0.08081 | 0.006378 | 0.845075 | 7.79E-06 | 8293 | 0.002405 | 19.98387 |
| rs74330453 | C | A | C | A | -0.21872 | 0.155252 | 0.102997 | 1.18E-06 | 8293 | 0.002838 | 23.59922 |
| rs76366817 | T | G | T | G | -0.43949 | 0.233901 | 0.001422 | 9.26E-07 | 8293 | 0.002895 | 24.06934 |
| rs7915131 | T | C | T | C | 0.059582 | -0.00799 | 0.737886 | 4.25E-06 | 8293 | 0.002544 | 21.14303 |
| rs9643828 | T | C | T | C | 0.065765 | -0.00748 | 0.763229 | 3.70E-06 | 8293 | 0.002576 | 21.40865 |
| rs9918422 | C | T | C | T | 0.341136 | 0.199399 | 0.29841 | 3.86E-07 | 8293 | 0.003097 | 25.75893 |
| GCST90199825 |  |  |  |  |  |  |  |  |  |  |  |
| SNP | effect_allele.exposure | other_allele.exposure | effect_allele.outcome | other_allele.outcome | beta.exposure | beta.outcome | pval.outcome | pval.exposure | samplesize.exposure | R2 | f |
| rs1150705 | G | T | G | T | -0.08112 | -0.02623 | 0.299702 | 5.00E-07 | 7920 | 0.00318 | 25.25733 |
| rs11635754 | G | T | G | T | -0.07716 | -0.00921 | 0.731742 | 8.19E-06 | 7920 | 0.002505 | 19.88724 |
| rs1253621 | A | G | A | G | -0.07466 | 0.006961 | 0.77936 | 5.00E-06 | 7920 | 0.002624 | 20.83211 |
| rs141346741 | G | C | G | C | -0.34745 | 0.25061 | 0.222686 | 9.55E-06 | 7920 | 0.002469 | 19.59478 |
| rs142710869 | C | A | C | A | -0.43154 | -0.03124 | 0.707123 | 2.76E-06 | 7920 | 0.002767 | 21.9679 |
| rs146878334 | G | A | G | A | -0.43223 | -0.11224 | 0.066368 | 9.48E-06 | 7920 | 0.00247 | 19.60765 |
| rs17368985 | C | T | C | T | -0.12813 | -0.06054 | 0.064594 | 8.01E-06 | 7920 | 0.002511 | 19.93075 |
| rs1885837 | C | T | C | T | -0.07159 | -0.00448 | 0.850203 | 6.38E-06 | 7920 | 0.002565 | 20.36469 |
| rs2298253 | C | T | C | T | -0.08803 | 0.009518 | 0.746583 | 9.12E-06 | 7920 | 0.00248 | 19.68151 |
| rs28377809 | A | G | A | G | -0.15019 | -0.13136 | 0.003207 | 8.22E-06 | 7920 | 0.002505 | 19.88081 |
| rs334125 | G | T | G | T | -0.07243 | 0.012807 | 0.589054 | 8.35E-06 | 7920 | 0.002501 | 19.851 |
| rs34092232 | T | A | T | A | -0.33774 | -0.13117 | 0.123108 | 9.42E-06 | 7920 | 0.002472 | 19.61974 |
| rs494342 | A | T | A | T | 0.090729 | -0.00284 | 0.923952 | 2.18E-07 | 7920 | 0.00338 | 26.85674 |
| rs58781224 | A | G | A | G | 0.210292 | 0.04943 | 0.648207 | 5.46E-06 | 7920 | 0.002603 | 20.66428 |
| rs6546829 | G | C | G | C | -0.08802 | -0.02629 | 0.318752 | 3.32E-07 | 7920 | 0.003279 | 26.04636 |
| rs7085530 | C | A | C | A | -0.09215 | -0.05079 | 0.119051 | 9.04E-06 | 7920 | 0.002482 | 19.69974 |
| rs71480524 | G | A | G | A | -0.29123 | -0.07896 | 0.431315 | 4.72E-06 | 7920 | 0.002638 | 20.94169 |
| rs74485582 | T | C | T | C | 0.132094 | -0.00477 | 0.922265 | 4.99E-06 | 7920 | 0.002625 | 20.83606 |
| rs77207094 | C | T | C | T | 0.261142 | 0.164115 | 0.018148 | 8.84E-06 | 7920 | 0.002487 | 19.74155 |
| rs77770732 | G | A | G | A | -0.21505 | 0.029969 | 0.753678 | 6.97E-06 | 7920 | 0.002544 | 20.19597 |
| rs78056593 | T | C | T | C | -0.13965 | 0.027421 | 0.554424 | 5.22E-06 | 7920 | 0.002614 | 20.74798 |
| rs7869355 | T | C | T | C | -0.07876 | 0.005615 | 0.83151 | 5.93E-06 | 7920 | 0.002583 | 20.5053 |
| rs78720491 | C | T | C | T | -0.20415 | -0.05538 | 0.433962 | 4.31E-06 | 7920 | 0.00266 | 21.11777 |
| GCST90199924 |  |  |  |  |  |  |  |  |  |  |  |
| SNP | effect_allele.exposure | other_allele.exposure | effect_allele.outcome | other_allele.outcome | beta.exposure | beta.outcome | pval.outcome | pval.exposure | samplesize.exposure | R2 | f |
| rs10179520 | A | G | A | G | 0.085513 | 0.008471 | 0.723522 | 8.67E-06 | 5440 | 0.003624 | 19.77713 |
| rs10279978 | A | G | A | G | -0.09422 | 0.002915 | 0.909239 | 4.94E-06 | 5440 | 0.00382 | 20.85196 |
| rs11070219 | T | C | T | C | 0.088027 | -0.0202 | 0.411632 | 4.90E-06 | 5440 | 0.003823 | 20.86948 |
| rs112909685 | A | G | A | G | -0.34597 | 0.077188 | 0.486595 | 5.32E-06 | 5440 | 0.003794 | 20.71085 |
| rs113281912 | A | T | A | T | -0.27787 | -0.00323 | 0.966065 | 2.19E-06 | 5440 | 0.004105 | 22.4164 |
| rs115343247 | C | T | C | T | 0.344135 | 0.002048 | 0.989301 | 2.90E-07 | 5440 | 0.004814 | 26.30742 |
| rs11656324 | C | G | C | G | 0.105057 | -0.00197 | 0.939761 | 2.45E-06 | 5440 | 0.004065 | 22.19623 |
| rs117836792 | G | C | G | C | -0.28437 | -0.0717 | 0.366564 | 5.26E-06 | 5440 | 0.003798 | 20.73274 |
| rs12474137 | G | A | G | A | -0.10028 | 0.05913 | 0.017245 | 4.48E-07 | 5440 | 0.004661 | 25.46649 |
| rs1284407 | A | G | A | G | 0.090219 | 0.002469 | 0.918978 | 6.64E-06 | 5440 | 0.003717 | 20.28663 |
| rs1375791 | G | A | G | A | -0.11378 | -0.01412 | 0.665839 | 1.84E-06 | 5440 | 0.004166 | 22.75016 |
| rs138082641 | A | G | A | G | -0.65695 | 0.00598 | 0.925429 | 4.39E-06 | 5440 | 0.003861 | 21.07967 |
| rs138220800 | A | G | A | G | 0.423086 | 0.026115 | 0.753508 | 9.40E-06 | 5440 | 0.003595 | 19.62284 |
| rs139132726 | T | C | T | C | -0.54497 | 0.04057 | 0.762056 | 1.28E-06 | 5440 | 0.004293 | 23.44825 |
| rs139554333 | G | A | G | A | 0.200713 | -0.04864 | 0.394464 | 6.81E-06 | 5440 | 0.003708 | 20.23901 |
| rs143462072 | A | G | A | G | 0.494545 | -0.20273 | 0.02099 | 6.81E-07 | 5440 | 0.004514 | 24.65966 |
| rs148197771 | A | C | A | C | 0.506161 | -0.24029 | 0.076547 | 9.01E-06 | 5440 | 0.00361 | 19.70366 |
| rs1898707 | A | G | A | G | -0.13008 | 0.037286 | 0.191546 | 6.32E-07 | 5440 | 0.00454 | 24.80283 |
| rs191630977 | A | G | A | G | 0.314923 | 0.007043 | 0.945682 | 5.25E-06 | 5440 | 0.003799 | 20.73582 |
| rs1946084 | G | A | G | A | -0.09011 | -0.00091 | 0.970367 | 3.93E-06 | 5440 | 0.0039 | 21.29231 |
| rs2284011 | T | C | T | C | -0.14264 | 0.06443 | 0.042294 | 1.27E-06 | 5440 | 0.004296 | 23.46457 |
| rs286018 | G | C | G | C | 0.095028 | -0.01139 | 0.642214 | 2.73E-06 | 5440 | 0.004027 | 21.98995 |
| rs60185985 | A | G | A | G | -0.15659 | -0.0046 | 0.919627 | 7.21E-06 | 5440 | 0.003688 | 20.1299 |
| rs6662518 | T | C | T | C | 0.122299 | -0.058 | 0.07063 | 5.30E-06 | 5440 | 0.003796 | 20.71924 |
| rs6671682 | T | C | T | C | -0.155 | 0.003832 | 0.917568 | 9.24E-06 | 5440 | 0.003601 | 19.65453 |
| rs7022505 | T | C | T | C | 0.151638 | -0.0551 | 0.118374 | 2.86E-06 | 5440 | 0.004011 | 21.89878 |
| rs8043403 | G | C | G | C | 0.125215 | 0.016403 | 0.597422 | 2.03E-06 | 5440 | 0.004131 | 22.55515 |
| rs879815 | A | G | A | G | 0.115722 | 0.002795 | 0.92123 | 6.95E-07 | 5440 | 0.004507 | 24.61924 |
| GCST90199927 |  |  |  |  |  |  |  |  |  |  |  |
| SNP | effect_allele.exposure | other_allele.exposure | effect_allele.outcome | other_allele.outcome | beta.exposure | beta.outcome | pval.outcome | pval.exposure | samplesize.exposure | R2 | f |
| rs10980156 | T | A | T | A | -0.2693 | 0.139505 | 0.171473 | 1.19E-06 | 7518 | 0.003129 | 23.59109 |
| rs115097924 | G | A | G | A | -0.19685 | -0.0335 | 0.681052 | 7.03E-06 | 7518 | 0.002678 | 20.18075 |
| rs11689313 | C | G | C | G | -0.08036 | 0.019135 | 0.423648 | 2.77E-06 | 7518 | 0.002914 | 21.96624 |
| rs12138275 | A | G | A | G | -0.11327 | -0.00251 | 0.94599 | 2.54E-06 | 7518 | 0.002936 | 22.13102 |
| rs12210538 | G | A | G | A | 0.112893 | -0.02642 | 0.406175 | 2.29E-09 | 7518 | 0.004727 | 35.69652 |
| rs142103014 | A | G | A | G | 0.281801 | -0.1133 | 0.165768 | 9.23E-06 | 7518 | 0.002609 | 19.65845 |
| rs35260072 | C | A | C | A | 0.110493 | 0.007744 | 0.759913 | 3.09E-11 | 7518 | 0.005834 | 44.10815 |
| rs4148325 | T | C | T | C | 0.114335 | -0.01625 | 0.503444 | 3.96E-11 | 7518 | 0.005771 | 43.62334 |
| rs4547965 | A | G | A | G | -0.23351 | 0.099882 | 0.206873 | 7.76E-06 | 7518 | 0.002653 | 19.99143 |
| rs6100964 | C | T | C | T | 0.089095 | -0.03051 | 0.385686 | 5.06E-06 | 7518 | 0.002761 | 20.80754 |
| rs73957577 | T | C | T | C | -0.18601 | 0.138085 | 0.009586 | 1.39E-06 | 7518 | 0.003089 | 23.28545 |
| rs7460378 | G | A | G | A | -0.07727 | 0.03812 | 0.108843 | 2.88E-06 | 7518 | 0.002904 | 21.89233 |
| rs75695195 | G | A | G | A | -0.18523 | 0.029786 | 0.430831 | 1.26E-06 | 7518 | 0.003114 | 23.47624 |
| rs75906023 | T | G | T | G | -0.36286 | 0.000999 | 0.990353 | 5.57E-07 | 7518 | 0.003322 | 25.04993 |
| rs77887994 | G | A | G | A | 0.284917 | 0.004181 | 0.955602 | 2.62E-06 | 7518 | 0.002927 | 22.06718 |
| rs79305797 | A | G | A | G | 0.283038 | -0.00017 | 0.998866 | 4.11E-06 | 7518 | 0.002814 | 21.20652 |
| GCST90199966 |  |  |  |  |  |  |  |  |  |  |  |
| SNP | effect_allele.exposure | other_allele.exposure | effect_allele.outcome | other_allele.outcome | beta.exposure | beta.outcome | pval.outcome | pval.exposure | samplesize.exposure | R2 | f |
| rs10800439 | C | G | C | G | 0.069603 | -0.02823 | 0.24147 | 6.93E-06 | 8157 | 0.002472 | 20.20771 |
| rs111296862 | A | G | A | G | -0.25214 | 0.041807 | 0.601973 | 7.89E-06 | 8157 | 0.002442 | 19.95999 |
| rs117307244 | C | T | C | T | -0.26653 | -0.12121 | 0.660683 | 9.88E-06 | 8157 | 0.002389 | 19.52875 |
| rs11952631 | C | A | C | A | 0.133566 | 0.047264 | 0.220353 | 4.24E-06 | 8157 | 0.002586 | 21.14707 |
| rs1322131 | C | A | C | A | 0.071536 | 0.023098 | 0.330782 | 6.68E-06 | 8157 | 0.00248 | 20.2766 |
| rs13268364 | T | C | T | C | -0.08079 | 0.004105 | 0.886246 | 6.99E-06 | 8157 | 0.00247 | 20.19237 |
| rs144005917 | G | A | G | A | -0.27262 | 0.099784 | 0.522816 | 4.23E-06 | 8157 | 0.002587 | 21.15403 |
| rs1502744 | G | A | G | A | 0.06749 | -0.012 | 0.612214 | 9.90E-06 | 8157 | 0.002389 | 19.52552 |
| rs189545437 | G | A | G | A | -1.0049 | 0.19602 | 0.009674 | 5.92E-08 | 8157 | 0.00359 | 29.38311 |
| rs2212765 | A | G | A | G | 0.095528 | -0.05821 | 0.158009 | 2.39E-06 | 8157 | 0.002721 | 22.25131 |
| rs2996024 | C | T | C | T | -0.07724 | 0.013626 | 0.594254 | 5.81E-06 | 8157 | 0.002513 | 20.5437 |
| rs4445527 | A | T | A | T | 0.142154 | -0.0425 | 0.233905 | 3.16E-06 | 8157 | 0.002655 | 21.71106 |
| rs62130720 | A | G | A | G | 0.259784 | -0.10375 | 0.186607 | 6.96E-06 | 8157 | 0.002471 | 20.19951 |
| rs7174584 | G | C | G | C | 0.077424 | -0.02322 | 0.359744 | 6.74E-06 | 8157 | 0.002478 | 20.25935 |
| rs76668835 | T | C | T | C | 0.174243 | -0.05315 | 0.394113 | 3.00E-06 | 8157 | 0.002668 | 21.8133 |
| rs79879479 | C | T | C | T | 0.191841 | -0.04546 | 0.471545 | 4.20E-06 | 8157 | 0.002589 | 21.16685 |
| rs9291418 | G | A | G | A | 0.078024 | -0.01722 | 0.562124 | 9.55E-06 | 8157 | 0.002397 | 19.59431 |
| rs9428344 | T | C | T | C | -0.06952 | -0.00268 | 0.910755 | 9.56E-06 | 8157 | 0.002397 | 19.59271 |
| GCST90200260 |  |  |  |  |  |  |  |  |  |  |  |
| SNP | effect_allele.exposure | other_allele.exposure | effect_allele.outcome | other_allele.outcome | beta.exposure | beta.outcome | pval.outcome | pval.exposure | samplesize.exposure | R2 | f |
| rs10022238 | T | G | T | G | 0.080021 | 0.010291 | 0.703706 | 5.72E-06 | 7990 | 0.002569 | 20.57373 |
| rs10171471 | C | T | C | T | -0.08322 | -0.03229 | 0.241776 | 2.51E-06 | 7990 | 0.002766 | 22.15432 |
| rs11519068 | C | T | C | T | 0.105594 | 0.026848 | 0.302202 | 8.04E-07 | 7990 | 0.003038 | 24.34235 |
| rs117913606 | A | G | A | G | -0.11798 | -0.00123 | 0.97801 | 3.40E-06 | 7990 | 0.002693 | 21.57275 |
| rs1293417 | C | A | C | A | 0.150881 | 0.020184 | 0.599518 | 1.11E-07 | 7990 | 0.003514 | 28.17199 |
| rs138275602 | A | G | A | G | 0.191072 | 0.039434 | 0.511896 | 5.84E-06 | 7990 | 0.002564 | 20.53587 |
| rs140412827 | G | A | G | A | -0.28185 | -0.12022 | 0.045768 | 9.37E-06 | 7990 | 0.002452 | 19.63156 |
| rs150406380 | G | C | G | C | -0.39328 | -0.11214 | 0.604046 | 9.03E-06 | 7990 | 0.00246 | 19.70137 |
| rs150967138 | A | G | A | G | 0.330149 | -0.01893 | 0.91009 | 1.97E-06 | 7990 | 0.002823 | 22.61587 |
| rs191217354 | A | G | A | G | 0.436721 | 0.150819 | 0.586857 | 2.68E-06 | 7990 | 0.00275 | 22.027 |
| rs2131280 | A | G | A | G | 0.07488 | 0.029568 | 0.221469 | 4.11E-06 | 7990 | 0.002648 | 21.20735 |
| rs218547 | A | C | A | C | 0.076157 | -0.00092 | 0.970053 | 9.98E-06 | 7990 | 0.002437 | 19.51047 |
| rs35396326 | G | C | G | C | 0.07976 | -0.01435 | 0.59262 | 6.87E-06 | 7990 | 0.002525 | 20.22337 |
| rs72836123 | T | C | T | C | 0.2162 | 0.091042 | 0.469724 | 4.84E-06 | 7990 | 0.002609 | 20.89392 |
| rs72849177 | G | A | G | A | -0.26756 | -0.0019 | 0.982585 | 1.20E-06 | 7990 | 0.002942 | 23.56948 |
| rs77176196 | C | T | C | T | -0.26305 | -0.06803 | 0.254969 | 8.90E-06 | 7990 | 0.002464 | 19.72835 |
| rs77794438 | C | T | C | T | 0.125831 | 0.019099 | 0.63788 | 6.03E-06 | 7990 | 0.002557 | 20.47397 |
| rs7982530 | A | G | A | G | -0.08552 | -0.01302 | 0.607047 | 7.65E-06 | 7990 | 0.0025 | 20.01798 |
| GCST90200502 |  |  |  |  |  |  |  |  |  |  |  |
| SNP | effect_allele.exposure | other_allele.exposure | effect_allele.outcome | other_allele.outcome | beta.exposure | beta.outcome | pval.outcome | pval.exposure | samplesize.exposure | R2 | f |
| rs10762982 | T | C | T | C | -0.10257 | -0.00152 | 0.96534 | 9.60E-06 | 7461 | 0.002619 | 19.58363 |
| rs10770141 | G | A | G | A | -0.07732 | -0.05289 | 0.033916 | 1.77E-06 | 7461 | 0.00305 | 22.82309 |
| rs111875922 | T | C | T | C | 0.460651 | 0.001455 | 0.989527 | 1.11E-14 | 7461 | 0.007938 | 59.68224 |
| rs113927841 | C | T | C | T | 0.2577 | 0.085341 | 0.28049 | 5.14E-06 | 7461 | 0.002778 | 20.77968 |
| rs115094506 | T | C | T | C | 0.27096 | 0.222223 | 0.0732 | 2.91E-06 | 7461 | 0.002923 | 21.86799 |
| rs116938011 | T | C | T | C | -0.70591 | -0.06329 | 0.334666 | 1.34E-17 | 7461 | 0.009681 | 72.91734 |
| rs117985404 | A | G | A | G | 0.342342 | 0.307229 | 0.00708 | 3.04E-10 | 7461 | 0.005286 | 39.63486 |
| rs12325244 | C | T | C | T | 0.261772 | -0.09212 | 0.664598 | 9.80E-06 | 7461 | 0.002613 | 19.54478 |
| rs12438830 | C | G | C | G | 0.123199 | 0.084952 | 0.035571 | 8.99E-06 | 7461 | 0.002635 | 19.70906 |
| rs12923643 | A | G | A | G | 0.310182 | 0.183236 | 0.0054 | 1.37E-13 | 7461 | 0.007285 | 54.74032 |
| rs144245963 | T | C | T | C | 0.52022 | -0.07181 | 0.764149 | 9.33E-15 | 7461 | 0.007982 | 60.01539 |
| rs148060734 | G | A | G | A | 0.427156 | 0.065688 | 0.232295 | 1.93E-06 | 7461 | 0.003028 | 22.6558 |
| rs165656 | C | G | C | G | 0.236019 | 0.019815 | 0.402618 | 3.63E-51 | 7461 | 0.029451 | 226.3386 |
| rs17357891 | G | C | G | C | -0.0966 | -0.01044 | 0.735531 | 6.88E-06 | 7461 | 0.002704 | 20.22089 |
| rs17712069 | G | T | G | T | 0.132088 | 0.011634 | 0.766433 | 2.82E-06 | 7461 | 0.002931 | 21.92624 |
| rs2040801 | C | G | C | G | 0.432099 | 0.013231 | 0.896561 | 5.10E-06 | 7461 | 0.00278 | 20.79279 |
| rs2540018 | G | A | G | A | -0.08385 | -0.0688 | 0.00698 | 5.61E-06 | 7461 | 0.002756 | 20.61088 |
| rs29575 | A | T | A | T | -0.07453 | 0.016635 | 0.507103 | 8.85E-06 | 7461 | 0.002639 | 19.73885 |
| rs36123375 | T | G | T | G | 0.160472 | -0.04781 | 0.370747 | 2.52E-06 | 7461 | 0.00296 | 22.1468 |
| rs4649043 | A | T | A | T | 0.138429 | -0.05328 | 0.246752 | 1.69E-06 | 7461 | 0.003062 | 22.90985 |
| rs4837433 | T | A | T | A | -0.12769 | -0.02117 | 0.622588 | 5.17E-07 | 7461 | 0.003366 | 25.19435 |
| rs4963272 | G | A | G | A | 0.094937 | 0.020413 | 0.486202 | 5.62E-06 | 7461 | 0.002755 | 20.60809 |
| rs7199777 | C | T | C | T | 0.081772 | -0.03246 | 0.174594 | 4.37E-07 | 7461 | 0.003409 | 25.5167 |
| rs72720906 | A | G | A | G | -0.18454 | 0.059575 | 0.39895 | 8.16E-06 | 7461 | 0.00266 | 19.89524 |
| rs76062400 | A | G | A | G | 0.22254 | 0.05695 | 0.327324 | 6.17E-06 | 7461 | 0.002731 | 20.42842 |
| rs76274897 | T | C | T | C | 0.098345 | -0.00347 | 0.90047 | 2.61E-06 | 7461 | 0.002951 | 22.07953 |
| rs76470122 | T | C | T | C | -0.11976 | -0.03515 | 0.308816 | 6.82E-06 | 7461 | 0.002706 | 20.23869 |
| rs78157656 | A | G | A | G | -0.28223 | -0.09629 | 0.574692 | 6.72E-06 | 7461 | 0.00271 | 20.26663 |
| rs79252520 | T | C | T | C | 0.196791 | -0.00679 | 0.934657 | 9.13E-06 | 7461 | 0.002631 | 19.67909 |
| rs80339217 | C | G | C | G | -0.24521 | 0.149498 | 0.298475 | 2.14E-08 | 7461 | 0.004187 | 31.35906 |
| rs9525958 | T | C | T | C | 0.16556 | 0.037423 | 0.680954 | 3.41E-06 | 7461 | 0.002883 | 21.56434 |
| GCST90200596 |  |  |  |  |  |  |  |  |  |  |  |
| SNP | effect_allele.exposure | other_allele.exposure | effect_allele.outcome | other_allele.outcome | beta.exposure | beta.outcome | pval.outcome | pval.exposure | samplesize.exposure | R2 | f |
| rs10028378 | G | T | G | T | 0.102313 | -0.00272 | 0.932569 | 9.07E-06 | 6764 | 0.002904 | 19.69216 |
| rs113767846 | C | G | C | G | -0.15321 | 0.002515 | 0.953919 | 4.99E-06 | 6764 | 0.003072 | 20.83634 |
| rs115315671 | T | C | T | C | 0.219891 | -0.04402 | 0.53232 | 5.97E-06 | 6764 | 0.003021 | 20.49174 |
| rs11605606 | G | A | G | A | 0.142511 | 0.031697 | 0.523388 | 2.76E-06 | 6764 | 0.003239 | 21.97201 |
| rs143324202 | C | A | C | A | -0.35769 | -0.10063 | 0.351345 | 1.96E-06 | 6764 | 0.003335 | 22.62741 |
| rs143540836 | A | T | A | T | 0.400449 | 0.149391 | 0.496639 | 7.39E-06 | 6764 | 0.002961 | 20.08268 |
| rs146610520 | T | C | T | C | -0.4803 | -0.11672 | 0.298399 | 2.07E-06 | 6764 | 0.00332 | 22.5218 |
| rs149960281 | T | C | T | C | -0.37026 | -0.05898 | 0.603123 | 8.94E-06 | 6764 | 0.002908 | 19.72032 |
| rs150436560 | C | G | C | G | -0.26138 | -0.09189 | 0.073748 | 5.84E-06 | 6764 | 0.003028 | 20.53491 |
| rs17056397 | T | C | T | C | -0.18366 | -0.00313 | 0.940996 | 1.14E-06 | 6764 | 0.003488 | 23.67086 |
| rs17220663 | A | G | A | G | -0.17136 | -0.05911 | 0.293612 | 3.03E-06 | 6764 | 0.003212 | 21.78975 |
| rs2431798 | T | C | T | C | 0.086421 | 0.036662 | 0.186233 | 7.91E-06 | 6764 | 0.002942 | 19.95442 |
| rs2472297 | T | C | T | C | 0.103039 | 0.04237 | 0.120064 | 1.17E-07 | 6764 | 0.004132 | 28.05808 |
| rs28889073 | A | G | A | G | -0.1197 | -0.078 | 0.056375 | 6.80E-06 | 6764 | 0.002985 | 20.24364 |
| rs4416419 | C | G | C | G | 0.089422 | 0.02505 | 0.317839 | 7.98E-07 | 6764 | 0.003589 | 24.35466 |
| rs62337911 | G | A | G | A | -0.2045 | 0.007278 | 0.921755 | 2.52E-06 | 6764 | 0.003265 | 22.14727 |
| rs6503078 | T | G | T | G | 0.091385 | -0.02081 | 0.448468 | 7.16E-06 | 6764 | 0.00297 | 20.14491 |
| rs74049524 | T | G | T | G | 0.194391 | -0.04004 | 0.49902 | 7.15E-06 | 6764 | 0.00297 | 20.14624 |
| rs7555782 | G | A | G | A | -0.17281 | -0.04058 | 0.450645 | 4.97E-06 | 6764 | 0.003073 | 20.84236 |
| rs79278676 | C | T | C | T | 0.220462 | 0.0258 | 0.624944 | 4.86E-06 | 6764 | 0.003079 | 20.88716 |
| rs79823355 | G | A | G | A | -0.15436 | 0.03893 | 0.394408 | 4.92E-06 | 6764 | 0.003076 | 20.86255 |
| rs79919890 | T | C | T | C | -0.18964 | -0.1138 | 0.069651 | 7.42E-06 | 6764 | 0.00296 | 20.0758 |
| rs9502656 | A | G | A | G | -0.30048 | 0.077917 | 0.548815 | 6.80E-06 | 6764 | 0.002985 | 20.24195 |
| rs9892419 | A | G | A | G | -0.15782 | -0.02809 | 0.530822 | 1.88E-06 | 6764 | 0.003346 | 22.70291 |
| GCST90200612 |  |  |  |  |  |  |  |  |  |  |  |
| SNP | effect_allele.exposure | other_allele.exposure | effect_allele.outcome | other_allele.outcome | beta.exposure | beta.outcome | pval.outcome | pval.exposure | samplesize.exposure | R2 | f |
| rs111892724 | T | G | T | G | 0.342568 | 0.000398 | 0.998591 | 4.43E-06 | 5745 | 0.003654 | 21.06311 |
| rs116970885 | T | G | T | G | -0.33118 | -0.22029 | 0.08007 | 3.50E-06 | 5745 | 0.003732 | 21.51374 |
| rs117531849 | C | A | C | A | 0.405549 | 0.011151 | 0.961615 | 1.73E-07 | 5745 | 0.004732 | 27.30451 |
| rs13102710 | T | G | T | G | 0.102879 | -0.00517 | 0.856341 | 1.84E-06 | 5745 | 0.003945 | 22.74609 |
| rs17509933 | G | A | G | A | 0.113229 | 0.05543 | 0.103153 | 4.73E-06 | 5745 | 0.003633 | 20.9379 |
| rs187272247 | A | C | A | C | -0.37235 | 0.21061 | 0.15899 | 5.93E-07 | 5745 | 0.004322 | 24.92659 |
| rs192963379 | A | G | A | G | -0.43792 | -0.00311 | 0.973672 | 1.15E-06 | 5745 | 0.004102 | 23.65702 |
| rs2642864 | C | T | C | T | 0.138185 | -0.00914 | 0.810121 | 4.04E-06 | 5745 | 0.003684 | 21.23741 |
| rs4338341 | A | G | A | G | -0.29091 | -0.1086 | 0.09214 | 1.28E-06 | 5745 | 0.004067 | 23.45134 |
| rs609812 | C | T | C | T | 0.152459 | -0.03085 | 0.421795 | 8.50E-06 | 5745 | 0.003438 | 19.81463 |
| rs61238733 | C | T | C | T | 0.137039 | 0.006615 | 0.905657 | 9.03E-06 | 5745 | 0.003419 | 19.69989 |
| rs6142813 | A | G | A | G | -0.0929 | 0.023377 | 0.398455 | 6.93E-06 | 5745 | 0.003506 | 20.20411 |
| rs72720588 | T | C | T | C | -0.42474 | -0.09978 | 0.087914 | 9.09E-09 | 5745 | 0.005716 | 33.0144 |
| rs75285112 | T | C | T | C | 0.316536 | 0.038488 | 0.588563 | 3.96E-06 | 5745 | 0.003691 | 21.2763 |
| rs75577088 | A | G | A | G | -0.28793 | -0.04759 | 0.641636 | 5.44E-06 | 5745 | 0.003586 | 20.66785 |
| rs78579961 | T | C | T | C | 0.110785 | 0.022123 | 0.449546 | 4.46E-06 | 5745 | 0.003652 | 21.05024 |
| rs79060316 | T | C | T | C | 0.355705 | 0.01442 | 0.897657 | 3.29E-07 | 5745 | 0.004518 | 26.06382 |
| rs7923082 | T | C | T | C | 0.105138 | 0.046607 | 0.073099 | 2.66E-06 | 5745 | 0.003823 | 22.0395 |
| rs8022260 | G | A | G | A | -0.13593 | -0.0724 | 0.034199 | 2.92E-06 | 5745 | 0.003792 | 21.85754 |
| GCST90200738 |  |  |  |  |  |  |  |  |  |  |  |
| SNP | effect_allele.exposure | other_allele.exposure | effect_allele.outcome | other_allele.outcome | beta.exposure | beta.outcome | pval.outcome | pval.exposure | samplesize.exposure | R2 | f |
| rs112770800 | T | C | T | C | 0.348947 | -0.01293 | 0.821338 | 2.51E-06 | 4601 | 0.004792 | 22.14683 |
| rs11582657 | T | A | T | A | -0.10298 | 0.038144 | 0.125728 | 8.43E-06 | 4601 | 0.004293 | 19.82806 |
| rs118130338 | T | C | T | C | 0.35242 | -0.1964 | 0.025768 | 8.85E-06 | 4601 | 0.004273 | 19.73703 |
| rs1354034 | C | T | C | T | -0.13459 | 0.015034 | 0.562537 | 2.47E-10 | 4601 | 0.008631 | 40.03743 |
| rs148538710 | T | C | T | C | 0.471634 | -0.20458 | 0.067471 | 5.33E-06 | 4601 | 0.004482 | 20.70541 |
| rs28542022 | C | T | C | T | -0.25776 | 0.024646 | 0.752452 | 1.51E-06 | 4601 | 0.005002 | 23.11936 |
| rs3115883 | C | T | C | T | 0.110413 | 0.005725 | 0.819122 | 2.99E-06 | 4601 | 0.004721 | 21.81518 |
| rs34355717 | C | G | C | G | 0.180604 | -0.01073 | 0.862706 | 9.86E-06 | 4601 | 0.004229 | 19.52985 |
| rs35816551 | T | A | T | A | -0.12171 | -0.0579 | 0.034943 | 4.11E-06 | 4601 | 0.004589 | 21.20424 |
| rs55674581 | T | C | T | C | 0.143856 | 0.008784 | 0.835038 | 2.36E-06 | 4601 | 0.004818 | 22.26487 |
| rs6099273 | T | C | T | C | -0.11105 | -0.0451 | 0.080705 | 7.50E-06 | 4601 | 0.004341 | 20.05217 |
| rs6450872 | T | C | T | C | 0.104519 | 0.007978 | 0.742944 | 1.41E-06 | 4601 | 0.005031 | 23.25305 |
| rs6953067 | G | C | G | C | -0.12029 | 0.04773 | 0.088855 | 4.42E-06 | 4601 | 0.004559 | 21.06287 |
| rs72624714 | A | G | A | G | -0.15809 | -0.02398 | 0.486626 | 3.23E-07 | 4601 | 0.005642 | 26.09364 |
| rs72881059 | T | C | T | C | 0.457045 | -0.11385 | 0.023938 | 7.44E-06 | 4601 | 0.004344 | 20.06717 |
| rs7358578 | A | G | A | G | 0.098332 | -0.02922 | 0.224774 | 3.06E-06 | 4601 | 0.004711 | 21.76976 |
| rs75415333 | T | C | T | C | -0.3451 | -0.01835 | 0.886248 | 8.82E-06 | 4601 | 0.004274 | 19.74245 |
| rs7662251 | T | C | T | C | 0.098349 | 0.000456 | 0.985015 | 4.24E-06 | 4601 | 0.004576 | 21.14249 |
| rs78099894 | G | A | G | A | -0.25566 | 0.054222 | 0.344457 | 2.80E-06 | 4601 | 0.004748 | 21.94206 |
| rs79731454 | A | G | A | G | -0.30242 | 0.038558 | 0.607495 | 9.43E-07 | 4601 | 0.005198 | 24.03153 |
| rs80184468 | T | C | T | C | -0.17019 | 0.047573 | 0.114658 | 1.00E-06 | 4601 | 0.005173 | 23.91461 |
| rs867687 | G | A | G | A | 0.108335 | -0.02084 | 0.428681 | 7.87E-06 | 4601 | 0.004321 | 19.96005 |
| rs9389561 | G | A | G | A | 0.109071 | -0.02822 | 0.275856 | 7.25E-07 | 4601 | 0.005307 | 24.53647 |
| GCST90200793 |  |  |  |  |  |  |  |  |  |  |  |
| SNP | effect_allele.exposure | other_allele.exposure | effect_allele.outcome | other_allele.outcome | beta.exposure | beta.outcome | pval.outcome | pval.exposure | samplesize.exposure | R2 | f |
| rs10199322 | C | T | C | T | 0.170195 | 0.011959 | 0.831699 | 3.48E-06 | 7043 | 0.003048 | 21.52391 |
| rs10746684 | G | C | G | C | -0.09496 | 0.045349 | 0.114659 | 2.00E-06 | 7043 | 0.003197 | 22.5857 |
| rs11101389 | A | T | A | T | -0.09554 | 0.009656 | 0.72134 | 6.47E-07 | 7043 | 0.003504 | 24.76082 |
| rs111624890 | T | C | T | C | 0.192307 | 0.0407 | 0.454435 | 6.22E-06 | 7043 | 0.002891 | 20.41298 |
| rs114562444 | G | A | G | A | -0.3292 | 0.079777 | 0.704826 | 4.63E-06 | 7043 | 0.002971 | 20.98069 |
| rs117249153 | T | C | T | C | 0.215867 | -0.01157 | 0.828861 | 2.14E-06 | 7043 | 0.00318 | 22.46069 |
| rs12881071 | A | G | A | G | 0.08299 | -0.00318 | 0.904346 | 6.25E-06 | 7043 | 0.00289 | 20.40498 |
| rs142931943 | T | C | T | C | 0.359766 | -0.17873 | 0.156224 | 7.41E-06 | 7043 | 0.002844 | 20.07871 |
| rs146114829 | T | C | T | C | -0.38813 | 0.14502 | 0.218323 | 9.66E-06 | 7043 | 0.002772 | 19.57283 |
| rs181147464 | C | T | C | T | -0.16152 | 0.012749 | 0.792669 | 2.00E-06 | 7043 | 0.003197 | 22.58581 |
| rs190901176 | A | G | A | G | -0.52538 | -0.55682 | 0.483898 | 5.35E-06 | 7043 | 0.002931 | 20.70125 |
| rs1976162 | G | A | G | A | -0.081 | -0.01319 | 0.587596 | 6.86E-06 | 7043 | 0.002864 | 20.22522 |
| rs212100 | C | T | C | T | -0.20316 | 0.006798 | 0.838032 | 5.62E-19 | 7043 | 0.01112 | 79.176 |
| rs55971546 | T | C | T | C | -0.36358 | 0.117378 | 0.043337 | 1.71E-20 | 7043 | 0.012077 | 86.07357 |
| rs56335545 | C | T | C | T | 0.164794 | -0.06778 | 0.041398 | 1.99E-06 | 7043 | 0.003199 | 22.59616 |
| rs57743625 | A | G | A | G | -0.18578 | 0.028264 | 0.277374 | 7.70E-17 | 7043 | 0.009769 | 69.4646 |
| rs61987308 | A | G | A | G | -0.16593 | 0.06578 | 0.13716 | 2.24E-06 | 7043 | 0.003168 | 22.37474 |
| rs6496949 | G | A | G | A | -0.07577 | -0.01483 | 0.531366 | 6.30E-06 | 7043 | 0.002887 | 20.38964 |
| rs74964662 | G | A | G | A | -0.24004 | 0.100435 | 0.167178 | 3.19E-06 | 7043 | 0.003071 | 21.69106 |
| rs74971002 | A | T | A | T | -0.13185 | 0.003314 | 0.936538 | 5.05E-06 | 7043 | 0.002947 | 20.81115 |
| rs76577804 | A | G | A | G | -0.19276 | -0.00596 | 0.934876 | 3.90E-06 | 7043 | 0.003017 | 21.30819 |
| rs78282920 | A | G | A | G | 0.120533 | -0.0149 | 0.671483 | 8.51E-06 | 7043 | 0.002806 | 19.81453 |
| rs79383019 | T | A | T | A | 0.20268 | -0.07454 | 0.327432 | 9.70E-06 | 7043 | 0.002771 | 19.56446 |
| rs9611880 | T | C | T | C | 0.14731 | 0.005042 | 0.875764 | 3.17E-07 | 7043 | 0.003699 | 26.1382 |
| rs9818127 | C | T | C | T | -0.14835 | 0.010405 | 0.831241 | 2.05E-06 | 7043 | 0.003192 | 22.54405 |
| GCST90200845 |  |  |  |  |  |  |  |  |  |  |  |
| SNP | effect_allele.exposure | other_allele.exposure | effect_allele.outcome | other_allele.outcome | beta.exposure | beta.outcome | pval.outcome | pval.exposure | samplesize.exposure | R2 | f |
| rs112368051 | T | C | T | C | 0.19648 | 0.093598 | 0.150242 | 5.18E-06 | 8193 | 0.002528 | 20.7628 |
| rs112885835 | G | C | G | C | 0.202285 | -0.00541 | 0.915744 | 6.52E-06 | 8193 | 0.002475 | 20.32503 |
| rs114357703 | A | G | A | G | 0.168056 | 0.076115 | 0.295805 | 8.88E-06 | 8193 | 0.002403 | 19.73354 |
| rs117592663 | A | G | A | G | 0.328736 | 0.159762 | 0.139947 | 6.99E-06 | 8193 | 0.002459 | 20.18982 |
| rs118103311 | C | T | C | T | -0.27773 | 0.023584 | 0.823485 | 1.42E-06 | 8193 | 0.002831 | 23.2508 |
| rs118182258 | A | G | A | G | 0.234681 | -0.08952 | 0.183139 | 1.15E-06 | 8193 | 0.002879 | 23.64798 |
| rs12597173 | C | G | C | G | -0.1012 | -0.02614 | 0.453644 | 8.77E-06 | 8193 | 0.002406 | 19.75807 |
| rs139721493 | T | C | T | C | 0.256059 | -0.06414 | 0.479002 | 5.79E-07 | 8193 | 0.00304 | 24.97643 |
| rs1401685 | C | A | C | A | -0.23661 | -0.05283 | 0.620688 | 4.49E-06 | 8193 | 0.002562 | 21.03916 |
| rs2348757 | C | T | C | T | -0.07727 | -0.01012 | 0.69768 | 5.45E-06 | 8193 | 0.002517 | 20.66873 |
| rs2505722 | T | G | T | G | -0.07732 | -0.00513 | 0.827916 | 7.09E-07 | 8193 | 0.002992 | 24.58453 |
| rs4643516 | G | C | G | C | 0.07663 | 0.040532 | 0.10247 | 6.40E-06 | 8193 | 0.00248 | 20.3609 |
| rs60110084 | G | A | G | A | -0.14278 | 0.0122 | 0.823329 | 6.14E-06 | 8193 | 0.002489 | 20.43863 |
| rs6590821 | C | T | C | T | 0.069928 | 0.01929 | 0.417103 | 5.55E-06 | 8193 | 0.002513 | 20.63249 |
| rs6993770 | T | A | T | A | -0.0758 | -0.01388 | 0.626216 | 9.83E-06 | 8193 | 0.00238 | 19.54014 |
| rs7490087 | A | C | A | C | -0.07571 | -0.0291 | 0.233039 | 2.48E-06 | 8193 | 0.0027 | 22.17849 |
| rs75039646 | A | C | A | C | 0.13916 | 0.130051 | 0.014932 | 8.10E-06 | 8193 | 0.002425 | 19.91047 |
| rs75060966 | A | G | A | G | 0.166759 | 0.100336 | 0.157707 | 2.39E-06 | 8193 | 0.002708 | 22.24386 |
| rs75562045 | A | G | A | G | -0.23198 | -0.07063 | 0.438555 | 8.71E-06 | 8193 | 0.002408 | 19.76992 |
| rs76097375 | T | A | T | A | -0.11998 | -0.08008 | 0.030576 | 8.94E-06 | 8193 | 0.002402 | 19.72075 |
| rs7642825 | A | G | A | G | -0.15098 | -0.01301 | 0.717621 | 6.77E-06 | 8193 | 0.002467 | 20.25357 |
| rs79857089 | C | G | C | G | -0.17376 | -0.09147 | 0.057512 | 2.93E-06 | 8193 | 0.002661 | 21.85704 |
