# Supplementary material for: The Negative Feedback of the Glutamine/Prostatitis Loop Identified Among 1400 Metabolites and Prostatitis via Mendelian Randomization
Source: Mediators Inflamm. 2025 Jul 15;2025:9648279. doi: 10.1155/mi/9648279 (PMC12283193; doi:10.1155/mi/9648279)
Supplement: Supporting Information 4 — Table S3. Setting conditions for instrumental variables (IVs) and results of heterogeneity and pleiotropy for the causality of prostatitis susceptibility to metabolites. [file 9648279.f4.docx]

**Table S3**. Setting conditions for instrumental variables (IVs) and results of heterogeneity and pleiotropy for the causality of prostatitis susceptibility to metabolites;

| Exposure | Outcome | P-value threshold | Linkage disequilibrium threshold | Heterogeneity (Q_pval) | Pleiotropy (pval) |
| --- | --- | --- | --- | --- | --- |
| finn-b-N14_PROSTATITIS  (Prostatitis) | GCST90199736  (Alpha-hydroxyisovalerate levels) | 1e-5 | clump_kb=10000, clump_r2=0.001 | 0.418 | **0.010** |
| finn-b-N14_PROSTATITIS  (Prostatitis) | GCST90199782  (Glutamine degradant levels) | 1e-5 | clump_kb=10000, clump_r2=0.001 | 0.285 | 0.486 |
| finn-b-N14_PROSTATITIS  (Prostatitis) | GCST90199825  (Pyrraline levels) | 1e-5 | clump_kb=10000, clump_r2=0.001 | 0.998 | 0.851 |
| finn-b-N14_PROSTATITIS  (Prostatitis) | GCST90199924  (N-methyltaurine levels) | 1e-5 | clump_kb=10000, clump_r2=0.001 | 0.262 | 0.081 |
| finn-b-N14_PROSTATITIS  (Prostatitis) | GCST90199927  (Histidine betaine (hercynine) levels) | 1e-5 | clump_kb=10000, clump_r2=0.001 | 0.341 | 0.074 |
| finn-b-N14_PROSTATITIS  (Prostatitis) | GCST90199966  (1-(1-enyl-oleoyl)-GPE (p-18:1) levels) | 1e-5 | clump_kb=10000, clump_r2=0.001 | 0.656 | 0.462 |
| finn-b-N14_PROSTATITIS  (Prostatitis) | GCST90200260  (Branched chain 14:0 dicarboxylic acid levels) | 1e-5 | clump_kb=10000, clump_r2=0.001 | 0.357 | 0.795 |
| finn-b-N14_PROSTATITIS  (Prostatitis) | GCST90200502  (X-12707 levels) | 1e-5 | clump_kb=10000, clump_r2=0.001 | 0.675 | 0.366 |
| finn-b-N14_PROSTATITIS  (Prostatitis) | GCST90200596  (X-23655 levels) | 1e-5 | clump_kb=10000, clump_r2=0.001 | 0.218 | 0.536 |
| finn-b-N14_PROSTATITIS  (Prostatitis) | GCST90200612  (X-23678 levels) | 1e-5 | clump_kb=10000, clump_r2=0.001 | 0.477 | 0.869 |
| finn-b-N14_PROSTATITIS  (Prostatitis) | GCST90200738  (AMP to IMP ratio) | 1e-5 | clump_kb=10000, clump_r2=0.001 | 0.275 | 0.899 |
| finn-b-N14_PROSTATITIS  (Prostatitis) | GCST90200793  (Glycolithocholate to glycolithocholate sulfate ratio) | 1e-5 | clump_kb=10000, clump_r2=0.001 | 0.541 | 0.315 |
| finn-b-N14_PROSTATITIS  (Prostatitis) | GCST90200845  (AMP to citrate ratio) | 1e-5 | clump_kb=10000, clump_r2=0.001 | 0.214 | 0.968 |
