# Supplementary material for: The Negative Feedback of the Glutamine/Prostatitis Loop Identified Among 1400 Metabolites and Prostatitis via Mendelian Randomization
Source: Mediators Inflamm. 2025 Jul 15;2025:9648279. doi: 10.1155/mi/9648279 (PMC12283193; doi:10.1155/mi/9648279)
Supplement: Supporting Information 5 — Table S4. The harmonized data for the causality of prostatitis susceptibility to metabolites. [file 9648279.f5.docx]

**Table S4**. The harmonized data for the causality of prostatitis susceptibility to metabolites;

| GCST90199736 |  |  |  |  |  |  |  |  |  |  |  |
| --- | --- | --- | --- | --- | --- | --- | --- | --- | --- | --- | --- |
| SNP | effect_allele.exposure | other_allele.exposure | effect_allele.outcome | other_allele.outcome | beta.exposure | beta.outcome | pval.outcome | pval.exposure | samplesize.exposure | R2 | f |
| rs10506840 | A | G | A | G | 0.158029 | -0.033 | 0.187026 | 2.91E-06 | 74658 | 0.000293 | 21.87509 |
| rs10509356 | T | C | T | C | -0.16399 | 0.005289 | 0.807682 | 8.85E-06 | 74658 | 0.000264 | 19.74525 |
| rs10964532 | A | T | A | T | 0.272128 | -0.00611 | 0.842831 | 3.29E-06 | 74658 | 0.00029 | 21.63615 |
| rs11219416 | T | G | T | G | -0.10841 | 0.019977 | 0.176261 | 7.28E-06 | 74658 | 0.000269 | 20.11795 |
| rs113296456 | A | G | A | G | -0.26343 | -0.00186 | 0.963747 | 7.14E-06 | 74658 | 0.00027 | 20.1541 |
| rs114303496 | C | T | C | T | -0.3852 | -0.0013 | 0.976409 | 9.80E-06 | 74658 | 0.000262 | 19.54883 |
| rs12616214 | C | A | C | A | 0.22652 | -0.05202 | 0.21733 | 4.19E-06 | 74658 | 0.000284 | 21.17472 |
| rs139043235 | T | C | T | C | -0.32289 | -0.09175 | 0.271567 | 2.85E-06 | 74658 | 0.000293 | 21.9156 |
| rs143907423 | T | C | T | C | 0.35087 | -0.09144 | 0.08948 | 4.22E-07 | 74658 | 0.000343 | 25.58985 |
| rs146456529 | TA | T | TA | T | -0.33882 | -0.0035 | 0.941795 | 5.20E-06 | 74658 | 0.000278 | 20.76172 |
| rs148804134 | G | A | G | A | -0.53198 | -0.08619 | 0.134414 | 1.88E-06 | 74658 | 0.000304 | 22.71446 |
| rs16895696 | T | C | T | C | -0.28421 | -0.00251 | 0.94455 | 1.36E-06 | 74658 | 0.000312 | 23.33123 |
| rs183401742 | T | C | T | C | -0.92138 | -0.08363 | 0.18166 | 1.78E-06 | 74658 | 0.000306 | 22.81669 |
| rs190907147 | G | A | G | A | -0.6221 | -0.07906 | 0.284035 | 4.26E-06 | 74658 | 0.000283 | 21.14529 |
| rs201254397 | C | T | C | T | 0.24565 | -0.03513 | 0.353101 | 5.49E-06 | 74658 | 0.000277 | 20.65602 |
| rs3017718 | T | C | T | C | 0.136757 | -0.00624 | 0.685811 | 5.61E-07 | 74658 | 0.000335 | 25.03978 |
| rs384810 | T | G | T | G | 0.130287 | -0.01314 | 0.468754 | 2.67E-06 | 74658 | 0.000295 | 22.03967 |
| rs60588618 | T | A | T | A | 0.167826 | 0.031426 | 0.105056 | 9.64E-07 | 74658 | 0.000321 | 23.99759 |
| rs629847 | G | A | G | A | 0.213482 | -0.01328 | 0.539227 | 5.18E-07 | 74658 | 0.000337 | 25.1945 |
| rs71509882 | A | C | A | C | -0.19259 | 0.001694 | 0.941985 | 6.85E-06 | 74658 | 0.000271 | 20.23395 |
| rs73165232 | T | A | T | A | 0.155215 | -0.01035 | 0.628144 | 2.66E-08 | 74658 | 0.000414 | 30.93818 |
| rs74691333 | C | T | C | T | -0.51166 | 0.015201 | 0.741245 | 8.19E-06 | 74658 | 0.000266 | 19.89172 |
| rs7841264 | T | C | T | C | -0.14929 | 0.035274 | 0.079092 | 1.25E-06 | 74658 | 0.000315 | 23.50317 |
| rs79095702 | A | T | A | T | 0.170667 | -0.03933 | 0.284277 | 5.51E-06 | 74658 | 0.000277 | 20.64899 |
| rs80320456 | A | G | A | G | 0.269186 | -0.05366 | 0.209272 | 7.31E-06 | 74658 | 0.000269 | 20.10904 |
| rs9933507 | C | T | C | T | 0.118646 | 0.014608 | 0.324133 | 6.01E-07 | 74658 | 0.000334 | 24.90751 |
| GCST90199782 |  |  |  |  |  |  |  |  |  |  |  |
| SNP | effect_allele.exposure | other_allele.exposure | effect_allele.outcome | other_allele.outcome | beta.exposure | beta.outcome | pval.outcome | pval.exposure | samplesize.exposure | R2 | f |
| rs10506840 | A | G | A | G | 0.158029 | -0.00108 | 0.960652 | 2.91E-06 | 74658 | 0.000293 | 21.87509 |
| rs10509356 | T | C | T | C | -0.16399 | -0.02225 | 0.238672 | 8.85E-06 | 74658 | 0.000264 | 19.74525 |
| rs10964532 | A | T | A | T | 0.272128 | 0.057617 | 0.031857 | 3.29E-06 | 74658 | 0.00029 | 21.63615 |
| rs11219416 | T | G | T | G | -0.10841 | -0.03253 | 0.011573 | 7.28E-06 | 74658 | 0.000269 | 20.11795 |
| rs113296456 | A | G | A | G | -0.26343 | -0.06929 | 0.052486 | 7.14E-06 | 74658 | 0.00027 | 20.1541 |
| rs114303496 | C | T | C | T | -0.3852 | -0.01307 | 0.732029 | 9.80E-06 | 74658 | 0.000262 | 19.54883 |
| rs12616214 | C | A | C | A | 0.22652 | -0.01726 | 0.637836 | 4.19E-06 | 74658 | 0.000284 | 21.17472 |
| rs139043235 | T | C | T | C | -0.32289 | -0.12902 | 0.077668 | 2.85E-06 | 74658 | 0.000293 | 21.9156 |
| rs143907423 | T | C | T | C | 0.35087 | -0.06521 | 0.16696 | 4.22E-07 | 74658 | 0.000343 | 25.58985 |
| rs146456529 | TA | T | TA | T | -0.33882 | -0.04187 | 0.317696 | 5.20E-06 | 74658 | 0.000278 | 20.76172 |
| rs148804134 | G | A | G | A | -0.53198 | -0.03821 | 0.448876 | 1.88E-06 | 74658 | 0.000304 | 22.71446 |
| rs16895696 | T | C | T | C | -0.28421 | -0.00369 | 0.906557 | 1.36E-06 | 74658 | 0.000312 | 23.33123 |
| rs183401742 | T | C | T | C | -0.92138 | -0.08235 | 0.129358 | 1.78E-06 | 74658 | 0.000306 | 22.81669 |
| rs190907147 | G | A | G | A | -0.6221 | 0.037933 | 0.557538 | 4.26E-06 | 74658 | 0.000283 | 21.14529 |
| rs201254397 | C | T | C | T | 0.24565 | 0.000589 | 0.985759 | 5.49E-06 | 74658 | 0.000277 | 20.65602 |
| rs3017718 | T | C | T | C | 0.136757 | 0.002482 | 0.85366 | 5.61E-07 | 74658 | 0.000335 | 25.03978 |
| rs384810 | T | G | T | G | 0.130287 | 0.007827 | 0.620841 | 2.67E-06 | 74658 | 0.000295 | 22.03967 |
| rs60588618 | T | A | T | A | 0.167826 | 0.013706 | 0.41814 | 9.64E-07 | 74658 | 0.000321 | 23.99759 |
| rs629847 | G | A | G | A | 0.213482 | 0.012216 | 0.517244 | 5.18E-07 | 74658 | 0.000337 | 25.1945 |
| rs71509882 | A | C | A | C | -0.19259 | 0.044107 | 0.02981 | 6.85E-06 | 74658 | 0.000271 | 20.23395 |
| rs73165232 | T | A | T | A | 0.155215 | 0.020534 | 0.270435 | 2.66E-08 | 74658 | 0.000414 | 30.93818 |
| rs74691333 | C | T | C | T | -0.51166 | 0.028918 | 0.468628 | 8.19E-06 | 74658 | 0.000266 | 19.89172 |
| rs7841264 | T | C | T | C | -0.14929 | -0.0027 | 0.877569 | 1.25E-06 | 74658 | 0.000315 | 23.50317 |
| rs79095702 | A | T | A | T | 0.170667 | -0.00675 | 0.832748 | 5.51E-06 | 74658 | 0.000277 | 20.64899 |
| rs80320456 | A | G | A | G | 0.269186 | 0.016266 | 0.662019 | 7.31E-06 | 74658 | 0.000269 | 20.10904 |
| rs9933507 | C | T | C | T | 0.118646 | 0.00416 | 0.74751 | 6.01E-07 | 74658 | 0.000334 | 24.90751 |
| GCST90199825 |  |  |  |  |  |  |  |  |  |  |  |
| SNP | effect_allele.exposure | other_allele.exposure | effect_allele.outcome | other_allele.outcome | beta.exposure | beta.outcome | pval.outcome | pval.exposure | samplesize.exposure | R2 | f |
| rs10506840 | A | G | A | G | 0.158029 | 0.027721 | 0.298702 | 2.91E-06 | 74658 | 0.000293 | 21.87509 |
| rs10509356 | T | C | T | C | -0.16399 | 0.01053 | 0.648537 | 8.85E-06 | 74658 | 0.000264 | 19.74525 |
| rs10964532 | A | T | A | T | 0.272128 | -0.01197 | 0.716381 | 3.29E-06 | 74658 | 0.00029 | 21.63615 |
| rs11219416 | T | G | T | G | -0.10841 | -0.01092 | 0.489397 | 7.28E-06 | 74658 | 0.000269 | 20.11795 |
| rs113296456 | A | G | A | G | -0.26343 | 0.023013 | 0.597468 | 7.14E-06 | 74658 | 0.00027 | 20.1541 |
| rs114303496 | C | T | C | T | -0.3852 | 0.014386 | 0.759238 | 9.80E-06 | 74658 | 0.000262 | 19.54883 |
| rs12616214 | C | A | C | A | 0.22652 | -0.02047 | 0.648476 | 4.19E-06 | 74658 | 0.000284 | 21.17472 |
| rs139043235 | T | C | T | C | -0.32289 | 0.022777 | 0.798528 | 2.85E-06 | 74658 | 0.000293 | 21.9156 |
| rs143907423 | T | C | T | C | 0.35087 | -0.0003 | 0.995791 | 4.22E-07 | 74658 | 0.000343 | 25.58985 |
| rs146456529 | TA | T | TA | T | -0.33882 | -0.0352 | 0.494546 | 5.20E-06 | 74658 | 0.000278 | 20.76172 |
| rs148804134 | G | A | G | A | -0.53198 | 0.028519 | 0.645208 | 1.88E-06 | 74658 | 0.000304 | 22.71446 |
| rs16895696 | T | C | T | C | -0.28421 | 0.046682 | 0.226594 | 1.36E-06 | 74658 | 0.000312 | 23.33123 |
| rs183401742 | T | C | T | C | -0.92138 | 0.032211 | 0.629002 | 1.78E-06 | 74658 | 0.000306 | 22.81669 |
| rs190907147 | G | A | G | A | -0.6221 | 0.032309 | 0.682755 | 4.26E-06 | 74658 | 0.000283 | 21.14529 |
| rs201254397 | C | T | C | T | 0.24565 | 0.006478 | 0.872621 | 5.49E-06 | 74658 | 0.000277 | 20.65602 |
| rs3017718 | T | C | T | C | 0.136757 | -0.00052 | 0.974968 | 5.61E-07 | 74658 | 0.000335 | 25.03978 |
| rs384810 | T | G | T | G | 0.130287 | 0.01034 | 0.593434 | 2.67E-06 | 74658 | 0.000295 | 22.03967 |
| rs60588618 | T | A | T | A | 0.167826 | -0.00146 | 0.943525 | 9.64E-07 | 74658 | 0.000321 | 23.99759 |
| rs629847 | G | A | G | A | 0.213482 | -0.02898 | 0.210144 | 5.18E-07 | 74658 | 0.000337 | 25.1945 |
| rs71509882 | A | C | A | C | -0.19259 | 0.03157 | 0.205415 | 6.85E-06 | 74658 | 0.000271 | 20.23395 |
| rs73165232 | T | A | T | A | 0.155215 | -0.03193 | 0.160924 | 2.66E-08 | 74658 | 0.000414 | 30.93818 |
| rs74691333 | C | T | C | T | -0.51166 | -0.06414 | 0.186993 | 8.19E-06 | 74658 | 0.000266 | 19.89172 |
| rs7841264 | T | C | T | C | -0.14929 | -0.00916 | 0.669035 | 1.25E-06 | 74658 | 0.000315 | 23.50317 |
| rs79095702 | A | T | A | T | 0.170667 | -0.01571 | 0.687258 | 5.51E-06 | 74658 | 0.000277 | 20.64899 |
| rs80320456 | A | G | A | G | 0.269186 | -0.01146 | 0.801118 | 7.31E-06 | 74658 | 0.000269 | 20.10904 |
| rs9933507 | C | T | C | T | 0.118646 | -0.00362 | 0.819302 | 6.01E-07 | 74658 | 0.000334 | 24.90751 |
| GCST90199924 |  |  |  |  |  |  |  |  |  |  |  |
| SNP | effect_allele.exposure | other_allele.exposure | effect_allele.outcome | other_allele.outcome | beta.exposure | beta.outcome | pval.outcome | pval.exposure | samplesize.exposure | R2 | f |
| rs10506840 | A | G | A | G | 0.158029 | 0.027507 | 0.393021 | 2.91E-06 | 74658 | 0.000293 | 21.87509 |
| rs10509356 | T | C | T | C | -0.16399 | -0.01463 | 0.597907 | 8.85E-06 | 74658 | 0.000264 | 19.74525 |
| rs10964532 | A | T | A | T | 0.272128 | 0.035998 | 0.367037 | 3.29E-06 | 74658 | 0.00029 | 21.63615 |
| rs11219416 | T | G | T | G | -0.10841 | -0.01982 | 0.294387 | 7.28E-06 | 74658 | 0.000269 | 20.11795 |
| rs113296456 | A | G | A | G | -0.26343 | 0.055701 | 0.28411 | 7.14E-06 | 74658 | 0.00027 | 20.1541 |
| rs114303496 | C | T | C | T | -0.3852 | 0.009796 | 0.863581 | 9.80E-06 | 74658 | 0.000262 | 19.54883 |
| rs12616214 | C | A | C | A | 0.22652 | -0.04033 | 0.454086 | 4.19E-06 | 74658 | 0.000284 | 21.17472 |
| rs139043235 | T | C | T | C | -0.32289 | 0.011348 | 0.913542 | 2.85E-06 | 74658 | 0.000293 | 21.9156 |
| rs143907423 | T | C | T | C | 0.35087 | 0.163975 | 0.020269 | 4.22E-07 | 74658 | 0.000343 | 25.58985 |
| rs146456529 | TA | T | TA | T | -0.33882 | -0.00673 | 0.91347 | 5.20E-06 | 74658 | 0.000278 | 20.76172 |
| rs148804134 | G | A | G | A | -0.53198 | 0.063739 | 0.38754 | 1.88E-06 | 74658 | 0.000304 | 22.71446 |
| rs16895696 | T | C | T | C | -0.28421 | 0.060343 | 0.201761 | 1.36E-06 | 74658 | 0.000312 | 23.33123 |
| rs183401742 | T | C | T | C | -0.92138 | 0.052895 | 0.51017 | 1.78E-06 | 74658 | 0.000306 | 22.81669 |
| rs190907147 | G | A | G | A | -0.6221 | 0.050084 | 0.590818 | 4.26E-06 | 74658 | 0.000283 | 21.14529 |
| rs201254397 | C | T | C | T | 0.24565 | -0.05623 | 0.246337 | 5.49E-06 | 74658 | 0.000277 | 20.65602 |
| rs3017718 | T | C | T | C | 0.136757 | 0.015644 | 0.431843 | 5.61E-07 | 74658 | 0.000335 | 25.03978 |
| rs384810 | T | G | T | G | 0.130287 | -0.03217 | 0.168719 | 2.67E-06 | 74658 | 0.000295 | 22.03967 |
| rs60588618 | T | A | T | A | 0.167826 | 0.008707 | 0.728249 | 9.64E-07 | 74658 | 0.000321 | 23.99759 |
| rs629847 | G | A | G | A | 0.213482 | 0.03918 | 0.156314 | 5.18E-07 | 74658 | 0.000337 | 25.1945 |
| rs71509882 | A | C | A | C | -0.19259 | 0.020251 | 0.495763 | 6.85E-06 | 74658 | 0.000271 | 20.23395 |
| rs73165232 | T | A | T | A | 0.155215 | 0.041945 | 0.122886 | 2.66E-08 | 74658 | 0.000414 | 30.93818 |
| rs74691333 | C | T | C | T | -0.51166 | -0.00799 | 0.889507 | 8.19E-06 | 74658 | 0.000266 | 19.89172 |
| rs7841264 | T | C | T | C | -0.14929 | 0.002981 | 0.908415 | 1.25E-06 | 74658 | 0.000315 | 23.50317 |
| rs79095702 | A | T | A | T | 0.170667 | 0.0825 | 0.078305 | 5.51E-06 | 74658 | 0.000277 | 20.64899 |
| rs80320456 | A | G | A | G | 0.269186 | 0.083198 | 0.12303 | 7.31E-06 | 74658 | 0.000269 | 20.10904 |
| rs9933507 | C | T | C | T | 0.118646 | 0.029618 | 0.118254 | 6.01E-07 | 74658 | 0.000334 | 24.90751 |
| GCST90199927 |  |  |  |  |  |  |  |  |  |  |  |
| SNP | effect_allele.exposure | other_allele.exposure | effect_allele.outcome | other_allele.outcome | beta.exposure | beta.outcome | pval.outcome | pval.exposure | samplesize.exposure | R2 | f |
| rs10506840 | A | G | A | G | 0.158029 | 0.032954 | 0.234966 | 2.91E-06 | 74658 | 0.000293 | 21.87509 |
| rs10509356 | T | C | T | C | -0.16399 | -0.04174 | 0.082579 | 8.85E-06 | 74658 | 0.000264 | 19.74525 |
| rs10964532 | A | T | A | T | 0.272128 | -0.05488 | 0.105207 | 3.29E-06 | 74658 | 0.00029 | 21.63615 |
| rs11219416 | T | G | T | G | -0.10841 | -0.02461 | 0.130281 | 7.28E-06 | 74658 | 0.000269 | 20.11795 |
| rs113296456 | A | G | A | G | -0.26343 | 0.058094 | 0.194349 | 7.14E-06 | 74658 | 0.00027 | 20.1541 |
| rs114303496 | C | T | C | T | -0.3852 | -0.00126 | 0.978899 | 9.80E-06 | 74658 | 0.000262 | 19.54883 |
| rs12616214 | C | A | C | A | 0.22652 | 0.034435 | 0.458709 | 4.19E-06 | 74658 | 0.000284 | 21.17472 |
| rs139043235 | T | C | T | C | -0.32289 | 0.010595 | 0.91164 | 2.85E-06 | 74658 | 0.000293 | 21.9156 |
| rs143907423 | T | C | T | C | 0.35087 | 0.078283 | 0.189235 | 4.22E-07 | 74658 | 0.000343 | 25.58985 |
| rs146456529 | TA | T | TA | T | -0.33882 | 0.031616 | 0.552591 | 5.20E-06 | 74658 | 0.000278 | 20.76172 |
| rs148804134 | G | A | G | A | -0.53198 | 0.00968 | 0.880894 | 1.88E-06 | 74658 | 0.000304 | 22.71446 |
| rs16895696 | T | C | T | C | -0.28421 | -0.07162 | 0.075694 | 1.36E-06 | 74658 | 0.000312 | 23.33123 |
| rs183401742 | T | C | T | C | -0.92138 | 0.012532 | 0.856066 | 1.78E-06 | 74658 | 0.000306 | 22.81669 |
| rs190907147 | G | A | G | A | -0.6221 | -0.06657 | 0.415843 | 4.26E-06 | 74658 | 0.000283 | 21.14529 |
| rs201254397 | C | T | C | T | 0.24565 | 0.060178 | 0.147397 | 5.49E-06 | 74658 | 0.000277 | 20.65602 |
| rs3017718 | T | C | T | C | 0.136757 | 0.033239 | 0.052253 | 5.61E-07 | 74658 | 0.000335 | 25.03978 |
| rs384810 | T | G | T | G | 0.130287 | -0.02467 | 0.219429 | 2.67E-06 | 74658 | 0.000295 | 22.03967 |
| rs60588618 | T | A | T | A | 0.167826 | -0.00886 | 0.679162 | 9.64E-07 | 74658 | 0.000321 | 23.99759 |
| rs629847 | G | A | G | A | 0.213482 | 0.024248 | 0.311601 | 5.18E-07 | 74658 | 0.000337 | 25.1945 |
| rs71509882 | A | C | A | C | -0.19259 | -0.00601 | 0.816453 | 6.85E-06 | 74658 | 0.000271 | 20.23395 |
| rs73165232 | T | A | T | A | 0.155215 | -0.00248 | 0.916052 | 2.66E-08 | 74658 | 0.000414 | 30.93818 |
| rs74691333 | C | T | C | T | -0.51166 | 0.001885 | 0.970583 | 8.19E-06 | 74658 | 0.000266 | 19.89172 |
| rs7841264 | T | C | T | C | -0.14929 | -0.04402 | 0.046641 | 1.25E-06 | 74658 | 0.000315 | 23.50317 |
| rs79095702 | A | T | A | T | 0.170667 | 0.031673 | 0.433769 | 5.51E-06 | 74658 | 0.000277 | 20.64899 |
| rs80320456 | A | G | A | G | 0.269186 | 0.001023 | 0.98279 | 7.31E-06 | 74658 | 0.000269 | 20.10904 |
| rs9933507 | C | T | C | T | 0.118646 | 0.014355 | 0.38099 | 6.01E-07 | 74658 | 0.000334 | 24.90751 |
| GCST90199966 |  |  |  |  |  |  |  |  |  |  |  |
| SNP | effect_allele.exposure | other_allele.exposure | effect_allele.outcome | other_allele.outcome | beta.exposure | beta.outcome | pval.outcome | pval.exposure | samplesize.exposure | R2 | f |
| rs10506840 | A | G | A | G | 0.158029 | 0.014629 | 0.574353 | 2.91E-06 | 74658 | 0.000293 | 21.87509 |
| rs10509356 | T | C | T | C | -0.16399 | 0.03413 | 0.131073 | 8.85E-06 | 74658 | 0.000264 | 19.74525 |
| rs10964532 | A | T | A | T | 0.272128 | -0.00164 | 0.959262 | 3.29E-06 | 74658 | 0.00029 | 21.63615 |
| rs11219416 | T | G | T | G | -0.10841 | -0.02791 | 0.07012 | 7.28E-06 | 74658 | 0.000269 | 20.11795 |
| rs113296456 | A | G | A | G | -0.26343 | -0.04611 | 0.279029 | 7.14E-06 | 74658 | 0.00027 | 20.1541 |
| rs114303496 | C | T | C | T | -0.3852 | 0.026888 | 0.556225 | 9.80E-06 | 74658 | 0.000262 | 19.54883 |
| rs12616214 | C | A | C | A | 0.22652 | 0.040155 | 0.359887 | 4.19E-06 | 74658 | 0.000284 | 21.17472 |
| rs139043235 | T | C | T | C | -0.32289 | 0.004327 | 0.960202 | 2.85E-06 | 74658 | 0.000293 | 21.9156 |
| rs143907423 | T | C | T | C | 0.35087 | 0.038885 | 0.488438 | 4.22E-07 | 74658 | 0.000343 | 25.58985 |
| rs146456529 | TA | T | TA | T | -0.33882 | 0.00274 | 0.956448 | 5.20E-06 | 74658 | 0.000278 | 20.76172 |
| rs148804134 | G | A | G | A | -0.53198 | 0.031599 | 0.598686 | 1.88E-06 | 74658 | 0.000304 | 22.71446 |
| rs16895696 | T | C | T | C | -0.28421 | -0.07328 | 0.051563 | 1.36E-06 | 74658 | 0.000312 | 23.33123 |
| rs183401742 | T | C | T | C | -0.92138 | 0.005467 | 0.932743 | 1.78E-06 | 74658 | 0.000306 | 22.81669 |
| rs190907147 | G | A | G | A | -0.6221 | 0.070178 | 0.364327 | 4.26E-06 | 74658 | 0.000283 | 21.14529 |
| rs201254397 | C | T | C | T | 0.24565 | -0.00075 | 0.984833 | 5.49E-06 | 74658 | 0.000277 | 20.65602 |
| rs3017718 | T | C | T | C | 0.136757 | -0.00503 | 0.754315 | 5.61E-07 | 74658 | 0.000335 | 25.03978 |
| rs384810 | T | G | T | G | 0.130287 | -0.03698 | 0.050387 | 2.67E-06 | 74658 | 0.000295 | 22.03967 |
| rs60588618 | T | A | T | A | 0.167826 | 0.018166 | 0.368637 | 9.64E-07 | 74658 | 0.000321 | 23.99759 |
| rs629847 | G | A | G | A | 0.213482 | 0.01888 | 0.402078 | 5.18E-07 | 74658 | 0.000337 | 25.1945 |
| rs71509882 | A | C | A | C | -0.19259 | -0.0211 | 0.384247 | 6.85E-06 | 74658 | 0.000271 | 20.23395 |
| rs73165232 | T | A | T | A | 0.155215 | 0.00922 | 0.678353 | 2.66E-08 | 74658 | 0.000414 | 30.93818 |
| rs74691333 | C | T | C | T | -0.51166 | -0.05111 | 0.283595 | 8.19E-06 | 74658 | 0.000266 | 19.89172 |
| rs7841264 | T | C | T | C | -0.14929 | -0.01485 | 0.477781 | 1.25E-06 | 74658 | 0.000315 | 23.50317 |
| rs79095702 | A | T | A | T | 0.170667 | -0.00209 | 0.956328 | 5.51E-06 | 74658 | 0.000277 | 20.64899 |
| rs80320456 | A | G | A | G | 0.269186 | 0.040784 | 0.357553 | 7.31E-06 | 74658 | 0.000269 | 20.10904 |
| rs9933507 | C | T | C | T | 0.118646 | 0.016451 | 0.286704 | 6.01E-07 | 74658 | 0.000334 | 24.90751 |
| GCST90200260 |  |  |  |  |  |  |  |  |  |  |  |
| SNP | effect_allele.exposure | other_allele.exposure | effect_allele.outcome | other_allele.outcome | beta.exposure | beta.outcome | pval.outcome | pval.exposure | samplesize.exposure | R2 | f |
| rs10506840 | A | G | A | G | 0.158029 | 0.022313 | 0.403103 | 2.91E-06 | 74658 | 0.000293 | 21.87509 |
| rs10509356 | T | C | T | C | -0.16399 | -0.03942 | 0.085933 | 8.85E-06 | 74658 | 0.000264 | 19.74525 |
| rs10964532 | A | T | A | T | 0.272128 | 0.014038 | 0.669137 | 3.29E-06 | 74658 | 0.00029 | 21.63615 |
| rs11219416 | T | G | T | G | -0.10841 | 0.007085 | 0.652224 | 7.28E-06 | 74658 | 0.000269 | 20.11795 |
| rs113296456 | A | G | A | G | -0.26343 | -0.0372 | 0.393722 | 7.14E-06 | 74658 | 0.00027 | 20.1541 |
| rs114303496 | C | T | C | T | -0.3852 | 0.070473 | 0.127386 | 9.80E-06 | 74658 | 0.000262 | 19.54883 |
| rs12616214 | C | A | C | A | 0.22652 | -0.05052 | 0.259158 | 4.19E-06 | 74658 | 0.000284 | 21.17472 |
| rs139043235 | T | C | T | C | -0.32289 | -0.02043 | 0.817818 | 2.85E-06 | 74658 | 0.000293 | 21.9156 |
| rs143907423 | T | C | T | C | 0.35087 | 0.143756 | 0.012237 | 4.22E-07 | 74658 | 0.000343 | 25.58985 |
| rs146456529 | TA | T | TA | T | -0.33882 | -0.02561 | 0.620643 | 5.20E-06 | 74658 | 0.000278 | 20.76172 |
| rs148804134 | G | A | G | A | -0.53198 | -0.03981 | 0.51434 | 1.88E-06 | 74658 | 0.000304 | 22.71446 |
| rs16895696 | T | C | T | C | -0.28421 | 0.003977 | 0.917901 | 1.36E-06 | 74658 | 0.000312 | 23.33123 |
| rs183401742 | T | C | T | C | -0.92138 | -0.0079 | 0.905199 | 1.78E-06 | 74658 | 0.000306 | 22.81669 |
| rs190907147 | G | A | G | A | -0.6221 | -0.00595 | 0.940079 | 4.26E-06 | 74658 | 0.000283 | 21.14529 |
| rs201254397 | C | T | C | T | 0.24565 | -0.00229 | 0.954558 | 5.49E-06 | 74658 | 0.000277 | 20.65602 |
| rs3017718 | T | C | T | C | 0.136757 | -0.00175 | 0.915098 | 5.61E-07 | 74658 | 0.000335 | 25.03978 |
| rs384810 | T | G | T | G | 0.130287 | -0.0124 | 0.521388 | 2.67E-06 | 74658 | 0.000295 | 22.03967 |
| rs60588618 | T | A | T | A | 0.167826 | -0.01924 | 0.35152 | 9.64E-07 | 74658 | 0.000321 | 23.99759 |
| rs629847 | G | A | G | A | 0.213482 | 0.018875 | 0.412955 | 5.18E-07 | 74658 | 0.000337 | 25.1945 |
| rs71509882 | A | C | A | C | -0.19259 | 0.036673 | 0.139898 | 6.85E-06 | 74658 | 0.000271 | 20.23395 |
| rs73165232 | T | A | T | A | 0.155215 | 0.034987 | 0.124414 | 2.66E-08 | 74658 | 0.000414 | 30.93818 |
| rs74691333 | C | T | C | T | -0.51166 | -0.0624 | 0.19948 | 8.19E-06 | 74658 | 0.000266 | 19.89172 |
| rs7841264 | T | C | T | C | -0.14929 | -0.02864 | 0.179373 | 1.25E-06 | 74658 | 0.000315 | 23.50317 |
| rs79095702 | A | T | A | T | 0.170667 | 0.064311 | 0.098934 | 5.51E-06 | 74658 | 0.000277 | 20.64899 |
| rs80320456 | A | G | A | G | 0.269186 | 0.05416 | 0.231007 | 7.31E-06 | 74658 | 0.000269 | 20.10904 |
| rs9933507 | C | T | C | T | 0.118646 | -0.00054 | 0.972743 | 6.01E-07 | 74658 | 0.000334 | 24.90751 |
| GCST90200502 |  |  |  |  |  |  |  |  |  |  |  |
| SNP | effect_allele.exposure | other_allele.exposure | effect_allele.outcome | other_allele.outcome | beta.exposure | beta.outcome | pval.outcome | pval.exposure | samplesize.exposure | R2 | f |
| rs10506840 | A | G | A | G | 0.158029 | 0.012071 | 0.649381 | 2.91E-06 | 74658 | 0.000293 | 21.87509 |
| rs10509356 | T | C | T | C | -0.16399 | -0.016 | 0.486621 | 8.85E-06 | 74658 | 0.000264 | 19.74525 |
| rs10964532 | A | T | A | T | 0.272128 | 0.007188 | 0.825229 | 3.29E-06 | 74658 | 0.00029 | 21.63615 |
| rs11219416 | T | G | T | G | -0.10841 | -0.0108 | 0.492055 | 7.28E-06 | 74658 | 0.000269 | 20.11795 |
| rs113296456 | A | G | A | G | -0.26343 | -0.06706 | 0.123196 | 7.14E-06 | 74658 | 0.00027 | 20.1541 |
| rs114303496 | C | T | C | T | -0.3852 | 0.038845 | 0.403248 | 9.80E-06 | 74658 | 0.000262 | 19.54883 |
| rs12616214 | C | A | C | A | 0.22652 | 0.007549 | 0.864067 | 4.19E-06 | 74658 | 0.000284 | 21.17472 |
| rs139043235 | T | C | T | C | -0.32289 | -0.13155 | 0.152063 | 2.85E-06 | 74658 | 0.000293 | 21.9156 |
| rs143907423 | T | C | T | C | 0.35087 | 0.003874 | 0.945735 | 4.22E-07 | 74658 | 0.000343 | 25.58985 |
| rs146456529 | TA | T | TA | T | -0.33882 | -0.05328 | 0.294074 | 5.20E-06 | 74658 | 0.000278 | 20.76172 |
| rs148804134 | G | A | G | A | -0.53198 | 0.058118 | 0.346527 | 1.88E-06 | 74658 | 0.000304 | 22.71446 |
| rs16895696 | T | C | T | C | -0.28421 | -0.00123 | 0.974729 | 1.36E-06 | 74658 | 0.000312 | 23.33123 |
| rs183401742 | T | C | T | C | -0.92138 | -0.00744 | 0.911466 | 1.78E-06 | 74658 | 0.000306 | 22.81669 |
| rs190907147 | G | A | G | A | -0.6221 | 0.039233 | 0.618823 | 4.26E-06 | 74658 | 0.000283 | 21.14529 |
| rs201254397 | C | T | C | T | 0.24565 | 0.075988 | 0.05787 | 5.49E-06 | 74658 | 0.000277 | 20.65602 |
| rs3017718 | T | C | T | C | 0.136757 | 0.001649 | 0.919895 | 5.61E-07 | 74658 | 0.000335 | 25.03978 |
| rs384810 | T | G | T | G | 0.130287 | 0.012956 | 0.501441 | 2.67E-06 | 74658 | 0.000295 | 22.03967 |
| rs60588618 | T | A | T | A | 0.167826 | 0.027272 | 0.186744 | 9.64E-07 | 74658 | 0.000321 | 23.99759 |
| rs629847 | G | A | G | A | 0.213482 | -0.00054 | 0.98119 | 5.18E-07 | 74658 | 0.000337 | 25.1945 |
| rs71509882 | A | C | A | C | -0.19259 | 0.038182 | 0.122672 | 6.85E-06 | 74658 | 0.000271 | 20.23395 |
| rs73165232 | T | A | T | A | 0.155215 | 0.041514 | 0.068673 | 2.66E-08 | 74658 | 0.000414 | 30.93818 |
| rs74691333 | C | T | C | T | -0.51166 | -0.0147 | 0.761089 | 8.19E-06 | 74658 | 0.000266 | 19.89172 |
| rs7841264 | T | C | T | C | -0.14929 | -0.00107 | 0.960094 | 1.25E-06 | 74658 | 0.000315 | 23.50317 |
| rs79095702 | A | T | A | T | 0.170667 | 0.05064 | 0.187311 | 5.51E-06 | 74658 | 0.000277 | 20.64899 |
| rs80320456 | A | G | A | G | 0.269186 | -0.01887 | 0.675879 | 7.31E-06 | 74658 | 0.000269 | 20.10904 |
| rs9933507 | C | T | C | T | 0.118646 | -0.01351 | 0.391223 | 6.01E-07 | 74658 | 0.000334 | 24.90751 |
| GCST90200596 |  |  |  |  |  |  |  |  |  |  |  |
| SNP | effect_allele.exposure | other_allele.exposure | effect_allele.outcome | other_allele.outcome | beta.exposure | beta.outcome | pval.outcome | pval.exposure | samplesize.exposure | R2 | f |
| rs10506840 | A | G | A | G | 0.158029 | -0.00501 | 0.861973 | 2.91E-06 | 74658 | 0.000293 | 21.87509 |
| rs10509356 | T | C | T | C | -0.16399 | 0.012003 | 0.630055 | 8.85E-06 | 74658 | 0.000264 | 19.74525 |
| rs10964532 | A | T | A | T | 0.272128 | -0.04487 | 0.207273 | 3.29E-06 | 74658 | 0.00029 | 21.63615 |
| rs11219416 | T | G | T | G | -0.10841 | 0.015931 | 0.350769 | 7.28E-06 | 74658 | 0.000269 | 20.11795 |
| rs113296456 | A | G | A | G | -0.26343 | -0.11083 | 0.019557 | 7.14E-06 | 74658 | 0.00027 | 20.1541 |
| rs114303496 | C | T | C | T | -0.3852 | -0.06101 | 0.230697 | 9.80E-06 | 74658 | 0.000262 | 19.54883 |
| rs12616214 | C | A | C | A | 0.22652 | 0.061767 | 0.208996 | 4.19E-06 | 74658 | 0.000284 | 21.17472 |
| rs139043235 | T | C | T | C | -0.32289 | -0.17845 | 0.064581 | 2.85E-06 | 74658 | 0.000293 | 21.9156 |
| rs143907423 | T | C | T | C | 0.35087 | 0.006253 | 0.921048 | 4.22E-07 | 74658 | 0.000343 | 25.58985 |
| rs146456529 | TA | T | TA | T | -0.33882 | -0.05201 | 0.352527 | 5.20E-06 | 74658 | 0.000278 | 20.76172 |
| rs148804134 | G | A | G | A | -0.53198 | 0.072152 | 0.289073 | 1.88E-06 | 74658 | 0.000304 | 22.71446 |
| rs16895696 | T | C | T | C | -0.28421 | 0.024529 | 0.567366 | 1.36E-06 | 74658 | 0.000312 | 23.33123 |
| rs183401742 | T | C | T | C | -0.92138 | -0.00114 | 0.987304 | 1.78E-06 | 74658 | 0.000306 | 22.81669 |
| rs190907147 | G | A | G | A | -0.6221 | -0.05319 | 0.519889 | 4.26E-06 | 74658 | 0.000283 | 21.14529 |
| rs201254397 | C | T | C | T | 0.24565 | 0.059066 | 0.172155 | 5.49E-06 | 74658 | 0.000277 | 20.65602 |
| rs3017718 | T | C | T | C | 0.136757 | -0.02638 | 0.140553 | 5.61E-07 | 74658 | 0.000335 | 25.03978 |
| rs384810 | T | G | T | G | 0.130287 | -0.01296 | 0.537406 | 2.67E-06 | 74658 | 0.000295 | 22.03967 |
| rs60588618 | T | A | T | A | 0.167826 | 0.046196 | 0.04027 | 9.64E-07 | 74658 | 0.000321 | 23.99759 |
| rs629847 | G | A | G | A | 0.213482 | 0.004067 | 0.870308 | 5.18E-07 | 74658 | 0.000337 | 25.1945 |
| rs71509882 | A | C | A | C | -0.19259 | 0.021282 | 0.431084 | 6.85E-06 | 74658 | 0.000271 | 20.23395 |
| rs73165232 | T | A | T | A | 0.155215 | 0.017607 | 0.475441 | 2.66E-08 | 74658 | 0.000414 | 30.93818 |
| rs74691333 | C | T | C | T | -0.51166 | 0.024141 | 0.642274 | 8.19E-06 | 74658 | 0.000266 | 19.89172 |
| rs7841264 | T | C | T | C | -0.14929 | 0.013633 | 0.557388 | 1.25E-06 | 74658 | 0.000315 | 23.50317 |
| rs79095702 | A | T | A | T | 0.170667 | -0.02374 | 0.580541 | 5.51E-06 | 74658 | 0.000277 | 20.64899 |
| rs80320456 | A | G | A | G | 0.269186 | 0.070879 | 0.149272 | 7.31E-06 | 74658 | 0.000269 | 20.10904 |
| rs9933507 | C | T | C | T | 0.118646 | 0.012901 | 0.453565 | 6.01E-07 | 74658 | 0.000334 | 24.90751 |
| GCST90200612 |  |  |  |  |  |  |  |  |  |  |  |
| SNP | effect_allele.exposure | other_allele.exposure | effect_allele.outcome | other_allele.outcome | beta.exposure | beta.outcome | pval.outcome | pval.exposure | samplesize.exposure | R2 | f |
| rs10506840 | A | G | A | G | 0.158029 | 0.042695 | 0.172588 | 2.91E-06 | 74658 | 0.000293 | 21.87509 |
| rs10509356 | T | C | T | C | -0.16399 | 0.012967 | 0.630404 | 8.85E-06 | 74658 | 0.000264 | 19.74525 |
| rs10964532 | A | T | A | T | 0.272128 | 0.017366 | 0.648155 | 3.29E-06 | 74658 | 0.00029 | 21.63615 |
| rs11219416 | T | G | T | G | -0.10841 | -0.02138 | 0.243718 | 7.28E-06 | 74658 | 0.000269 | 20.11795 |
| rs113296456 | A | G | A | G | -0.26343 | -0.05775 | 0.26119 | 7.14E-06 | 74658 | 0.00027 | 20.1541 |
| rs114303496 | C | T | C | T | -0.3852 | 0.073834 | 0.180433 | 9.80E-06 | 74658 | 0.000262 | 19.54883 |
| rs12616214 | C | A | C | A | 0.22652 | -0.02261 | 0.673232 | 4.19E-06 | 74658 | 0.000284 | 21.17472 |
| rs139043235 | T | C | T | C | -0.32289 | -0.22726 | 0.038567 | 2.85E-06 | 74658 | 0.000293 | 21.9156 |
| rs143907423 | T | C | T | C | 0.35087 | 0.12372 | 0.069451 | 4.22E-07 | 74658 | 0.000343 | 25.58985 |
| rs146456529 | TA | T | TA | T | -0.33882 | -0.06027 | 0.326906 | 5.20E-06 | 74658 | 0.000278 | 20.76172 |
| rs148804134 | G | A | G | A | -0.53198 | 0.04556 | 0.527221 | 1.88E-06 | 74658 | 0.000304 | 22.71446 |
| rs16895696 | T | C | T | C | -0.28421 | -0.00607 | 0.893717 | 1.36E-06 | 74658 | 0.000312 | 23.33123 |
| rs183401742 | T | C | T | C | -0.92138 | -0.05731 | 0.44075 | 1.78E-06 | 74658 | 0.000306 | 22.81669 |
| rs190907147 | G | A | G | A | -0.6221 | 0.144118 | 0.128923 | 4.26E-06 | 74658 | 0.000283 | 21.14529 |
| rs201254397 | C | T | C | T | 0.24565 | 0.035367 | 0.461363 | 5.49E-06 | 74658 | 0.000277 | 20.65602 |
| rs3017718 | T | C | T | C | 0.136757 | -0.00873 | 0.649461 | 5.61E-07 | 74658 | 0.000335 | 25.03978 |
| rs384810 | T | G | T | G | 0.130287 | 0.002864 | 0.899753 | 2.67E-06 | 74658 | 0.000295 | 22.03967 |
| rs60588618 | T | A | T | A | 0.167826 | -0.00252 | 0.917173 | 9.64E-07 | 74658 | 0.000321 | 23.99759 |
| rs629847 | G | A | G | A | 0.213482 | 0.004601 | 0.866472 | 5.18E-07 | 74658 | 0.000337 | 25.1945 |
| rs71509882 | A | C | A | C | -0.19259 | -0.02233 | 0.441547 | 6.85E-06 | 74658 | 0.000271 | 20.23395 |
| rs73165232 | T | A | T | A | 0.155215 | 0.000653 | 0.980606 | 2.66E-08 | 74658 | 0.000414 | 30.93818 |
| rs74691333 | C | T | C | T | -0.51166 | 0.027091 | 0.630719 | 8.19E-06 | 74658 | 0.000266 | 19.89172 |
| rs7841264 | T | C | T | C | -0.14929 | 0.009504 | 0.703712 | 1.25E-06 | 74658 | 0.000315 | 23.50317 |
| rs79095702 | A | T | A | T | 0.170667 | -0.06626 | 0.152221 | 5.51E-06 | 74658 | 0.000277 | 20.64899 |
| rs80320456 | A | G | A | G | 0.269186 | -0.0089 | 0.867639 | 7.31E-06 | 74658 | 0.000269 | 20.10904 |
| rs9933507 | C | T | C | T | 0.118646 | -0.02759 | 0.134545 | 6.01E-07 | 74658 | 0.000334 | 24.90751 |
| GCST90200738 |  |  |  |  |  |  |  |  |  |  |  |
| SNP | effect_allele.exposure | other_allele.exposure | effect_allele.outcome | other_allele.outcome | beta.exposure | beta.outcome | pval.outcome | pval.exposure | samplesize.exposure | R2 | f |
| rs10506840 | A | G | A | G | 0.158029 | 0.017157 | 0.627017 | 2.91E-06 | 74658 | 0.000293 | 21.87509 |
| rs10509356 | T | C | T | C | -0.16399 | 0.022798 | 0.45174 | 8.85E-06 | 74658 | 0.000264 | 19.74525 |
| rs10964532 | A | T | A | T | 0.272128 | 0.043372 | 0.318296 | 3.29E-06 | 74658 | 0.00029 | 21.63615 |
| rs11219416 | T | G | T | G | -0.10841 | -0.02443 | 0.239621 | 7.28E-06 | 74658 | 0.000269 | 20.11795 |
| rs113296456 | A | G | A | G | -0.26343 | 0.016572 | 0.772706 | 7.14E-06 | 74658 | 0.00027 | 20.1541 |
| rs114303496 | C | T | C | T | -0.3852 | -0.13486 | 0.030593 | 9.80E-06 | 74658 | 0.000262 | 19.54883 |
| rs12616214 | C | A | C | A | 0.22652 | -0.03255 | 0.582193 | 4.19E-06 | 74658 | 0.000284 | 21.17472 |
| rs139043235 | T | C | T | C | -0.32289 | -0.11919 | 0.29471 | 2.85E-06 | 74658 | 0.000293 | 21.9156 |
| rs143907423 | T | C | T | C | 0.35087 | 0.027418 | 0.732312 | 4.22E-07 | 74658 | 0.000343 | 25.58985 |
| rs146456529 | TA | T | TA | T | -0.33882 | -0.06697 | 0.314545 | 5.20E-06 | 74658 | 0.000278 | 20.76172 |
| rs148804134 | G | A | G | A | -0.53198 | 0.143759 | 0.087284 | 1.88E-06 | 74658 | 0.000304 | 22.71446 |
| rs16895696 | T | C | T | C | -0.28421 | 0.037211 | 0.469188 | 1.36E-06 | 74658 | 0.000312 | 23.33123 |
| rs183401742 | T | C | T | C | -0.92138 | -0.02799 | 0.749505 | 1.78E-06 | 74658 | 0.000306 | 22.81669 |
| rs190907147 | G | A | G | A | -0.6221 | 0.04741 | 0.637846 | 4.26E-06 | 74658 | 0.000283 | 21.14529 |
| rs201254397 | C | T | C | T | 0.24565 | 0.029163 | 0.574025 | 5.49E-06 | 74658 | 0.000277 | 20.65602 |
| rs3017718 | T | C | T | C | 0.136757 | -0.01621 | 0.455229 | 5.61E-07 | 74658 | 0.000335 | 25.03978 |
| rs384810 | T | G | T | G | 0.130287 | 0.056109 | 0.027152 | 2.67E-06 | 74658 | 0.000295 | 22.03967 |
| rs60588618 | T | A | T | A | 0.167826 | -0.01147 | 0.677313 | 9.64E-07 | 74658 | 0.000321 | 23.99759 |
| rs629847 | G | A | G | A | 0.213482 | -0.05155 | 0.087339 | 5.18E-07 | 74658 | 0.000337 | 25.1945 |
| rs71509882 | A | C | A | C | -0.19259 | 0.019818 | 0.548015 | 6.85E-06 | 74658 | 0.000271 | 20.23395 |
| rs73165232 | T | A | T | A | 0.155215 | -0.00887 | 0.770936 | 2.66E-08 | 74658 | 0.000414 | 30.93818 |
| rs74691333 | C | T | C | T | -0.51166 | -0.00547 | 0.932446 | 8.19E-06 | 74658 | 0.000266 | 19.89172 |
| rs7841264 | T | C | T | C | -0.14929 | -0.04623 | 0.105032 | 1.25E-06 | 74658 | 0.000315 | 23.50317 |
| rs79095702 | A | T | A | T | 0.170667 | 0.017918 | 0.729162 | 5.51E-06 | 74658 | 0.000277 | 20.64899 |
| rs80320456 | A | G | A | G | 0.269186 | -0.02372 | 0.682808 | 7.31E-06 | 74658 | 0.000269 | 20.10904 |
| rs9933507 | C | T | C | T | 0.118646 | -0.03175 | 0.128284 | 6.01E-07 | 74658 | 0.000334 | 24.90751 |
| GCST90200793 |  |  |  |  |  |  |  |  |  |  |  |
| SNP | effect_allele.exposure | other_allele.exposure | effect_allele.outcome | other_allele.outcome | beta.exposure | beta.outcome | pval.outcome | pval.exposure | samplesize.exposure | R2 | f |
| rs10506840 | A | G | A | G | 0.158029 | -0.04614 | 0.106487 | 2.91E-06 | 74658 | 0.000293 | 21.87509 |
| rs10509356 | T | C | T | C | -0.16399 | 0.024523 | 0.323326 | 8.85E-06 | 74658 | 0.000264 | 19.74525 |
| rs10964532 | A | T | A | T | 0.272128 | 0.020646 | 0.556623 | 3.29E-06 | 74658 | 0.00029 | 21.63615 |
| rs11219416 | T | G | T | G | -0.10841 | 0.005646 | 0.736679 | 7.28E-06 | 74658 | 0.000269 | 20.11795 |
| rs113296456 | A | G | A | G | -0.26343 | -0.04848 | 0.302993 | 7.14E-06 | 74658 | 0.00027 | 20.1541 |
| rs114303496 | C | T | C | T | -0.3852 | -0.02354 | 0.641659 | 9.80E-06 | 74658 | 0.000262 | 19.54883 |
| rs12616214 | C | A | C | A | 0.22652 | -0.06471 | 0.18018 | 4.19E-06 | 74658 | 0.000284 | 21.17472 |
| rs139043235 | T | C | T | C | -0.32289 | 0.133411 | 0.159588 | 2.85E-06 | 74658 | 0.000293 | 21.9156 |
| rs143907423 | T | C | T | C | 0.35087 | -0.01019 | 0.871032 | 4.22E-07 | 74658 | 0.000343 | 25.58985 |
| rs146456529 | TA | T | TA | T | -0.33882 | 0.135251 | 0.01557 | 5.20E-06 | 74658 | 0.000278 | 20.76172 |
| rs148804134 | G | A | G | A | -0.53198 | -0.00829 | 0.901925 | 1.88E-06 | 74658 | 0.000304 | 22.71446 |
| rs16895696 | T | C | T | C | -0.28421 | -0.03829 | 0.357247 | 1.36E-06 | 74658 | 0.000312 | 23.33123 |
| rs183401742 | T | C | T | C | -0.92138 | -0.04174 | 0.558027 | 1.78E-06 | 74658 | 0.000306 | 22.81669 |
| rs190907147 | G | A | G | A | -0.6221 | 0.077635 | 0.359371 | 4.26E-06 | 74658 | 0.000283 | 21.14529 |
| rs201254397 | C | T | C | T | 0.24565 | 0.03389 | 0.431298 | 5.49E-06 | 74658 | 0.000277 | 20.65602 |
| rs3017718 | T | C | T | C | 0.136757 | 0.010539 | 0.549592 | 5.61E-07 | 74658 | 0.000335 | 25.03978 |
| rs384810 | T | G | T | G | 0.130287 | -0.01413 | 0.492987 | 2.67E-06 | 74658 | 0.000295 | 22.03967 |
| rs60588618 | T | A | T | A | 0.167826 | -0.02151 | 0.328918 | 9.64E-07 | 74658 | 0.000321 | 23.99759 |
| rs629847 | G | A | G | A | 0.213482 | 0.011413 | 0.644819 | 5.18E-07 | 74658 | 0.000337 | 25.1945 |
| rs71509882 | A | C | A | C | -0.19259 | 0.031982 | 0.228019 | 6.85E-06 | 74658 | 0.000271 | 20.23395 |
| rs73165232 | T | A | T | A | 0.155215 | -0.01675 | 0.494865 | 2.66E-08 | 74658 | 0.000414 | 30.93818 |
| rs74691333 | C | T | C | T | -0.51166 | -0.03023 | 0.559268 | 8.19E-06 | 74658 | 0.000266 | 19.89172 |
| rs7841264 | T | C | T | C | -0.14929 | -0.026 | 0.255814 | 1.25E-06 | 74658 | 0.000315 | 23.50317 |
| rs79095702 | A | T | A | T | 0.170667 | 0.019263 | 0.646112 | 5.51E-06 | 74658 | 0.000277 | 20.64899 |
| rs80320456 | A | G | A | G | 0.269186 | -0.01691 | 0.727003 | 7.31E-06 | 74658 | 0.000269 | 20.10904 |
| rs9933507 | C | T | C | T | 0.118646 | -0.01367 | 0.417505 | 6.01E-07 | 74658 | 0.000334 | 24.90751 |
| GCST90200845 |  |  |  |  |  |  |  |  |  |  |  |
| SNP | effect_allele.exposure | other_allele.exposure | effect_allele.outcome | other_allele.outcome | beta.exposure | beta.outcome | pval.outcome | pval.exposure | samplesize.exposure | R2 | f |
| rs10506840 | A | G | A | G | 0.158029 | 0.038009 | 0.142341 | 2.91E-06 | 74658 | 0.000293 | 21.87509 |
| rs10509356 | T | C | T | C | -0.16399 | 0.037269 | 0.096965 | 8.85E-06 | 74658 | 0.000264 | 19.74525 |
| rs10964532 | A | T | A | T | 0.272128 | 0.001939 | 0.951747 | 3.29E-06 | 74658 | 0.00029 | 21.63615 |
| rs11219416 | T | G | T | G | -0.10841 | 0.003274 | 0.830756 | 7.28E-06 | 74658 | 0.000269 | 20.11795 |
| rs113296456 | A | G | A | G | -0.26343 | -0.00158 | 0.970381 | 7.14E-06 | 74658 | 0.00027 | 20.1541 |
| rs114303496 | C | T | C | T | -0.3852 | -0.04204 | 0.352899 | 9.80E-06 | 74658 | 0.000262 | 19.54883 |
| rs12616214 | C | A | C | A | 0.22652 | 0.0045 | 0.917848 | 4.19E-06 | 74658 | 0.000284 | 21.17472 |
| rs139043235 | T | C | T | C | -0.32289 | 0.040305 | 0.642355 | 2.85E-06 | 74658 | 0.000293 | 21.9156 |
| rs143907423 | T | C | T | C | 0.35087 | -0.08782 | 0.117664 | 4.22E-07 | 74658 | 0.000343 | 25.58985 |
| rs146456529 | TA | T | TA | T | -0.33882 | 0.045826 | 0.358417 | 5.20E-06 | 74658 | 0.000278 | 20.76172 |
| rs148804134 | G | A | G | A | -0.53198 | 0.02539 | 0.672566 | 1.88E-06 | 74658 | 0.000304 | 22.71446 |
| rs16895696 | T | C | T | C | -0.28421 | -0.05038 | 0.177296 | 1.36E-06 | 74658 | 0.000312 | 23.33123 |
| rs183401742 | T | C | T | C | -0.92138 | -0.01057 | 0.869697 | 1.78E-06 | 74658 | 0.000306 | 22.81669 |
| rs190907147 | G | A | G | A | -0.6221 | -0.08067 | 0.294217 | 4.26E-06 | 74658 | 0.000283 | 21.14529 |
| rs201254397 | C | T | C | T | 0.24565 | 0.060466 | 0.123425 | 5.49E-06 | 74658 | 0.000277 | 20.65602 |
| rs3017718 | T | C | T | C | 0.136757 | -0.01789 | 0.263751 | 5.61E-07 | 74658 | 0.000335 | 25.03978 |
| rs384810 | T | G | T | G | 0.130287 | -0.00694 | 0.712708 | 2.67E-06 | 74658 | 0.000295 | 22.03967 |
| rs60588618 | T | A | T | A | 0.167826 | 0.044931 | 0.025362 | 9.64E-07 | 74658 | 0.000321 | 23.99759 |
| rs629847 | G | A | G | A | 0.213482 | -0.01029 | 0.646336 | 5.18E-07 | 74658 | 0.000337 | 25.1945 |
| rs71509882 | A | C | A | C | -0.19259 | 0.036454 | 0.130648 | 6.85E-06 | 74658 | 0.000271 | 20.23395 |
| rs73165232 | T | A | T | A | 0.155215 | -0.02194 | 0.32167 | 2.66E-08 | 74658 | 0.000414 | 30.93818 |
| rs74691333 | C | T | C | T | -0.51166 | -0.01158 | 0.807442 | 8.19E-06 | 74658 | 0.000266 | 19.89172 |
| rs7841264 | T | C | T | C | -0.14929 | -0.00898 | 0.666512 | 1.25E-06 | 74658 | 0.000315 | 23.50317 |
| rs79095702 | A | T | A | T | 0.170667 | -0.00805 | 0.832577 | 5.51E-06 | 74658 | 0.000277 | 20.64899 |
| rs80320456 | A | G | A | G | 0.269186 | 0.039595 | 0.372909 | 7.31E-06 | 74658 | 0.000269 | 20.10904 |
| rs9933507 | C | T | C | T | 0.118646 | 0.033892 | 0.02725 | 6.01E-07 | 74658 | 0.000334 | 24.90751 |
